# Neonatal and adult recent thymic emigrants produce IL-8 and express complement receptors CR1 and CR2

**DOI:** 10.1172/jci.insight.93739

**Published:** 2017-08-17

**Authors:** Marcin L. Pekalski, Arcadio Rubio García, Ricardo C. Ferreira, Daniel B. Rainbow, Deborah J. Smyth, Meghavi Mashar, Jane Brady, Natalia Savinykh, Xaquin Castro Dopico, Sumiyya Mahmood, Simon Duley, Helen E. Stevens, Neil M. Walker, Antony J. Cutler, Frank Waldron-Lynch, David B. Dunger, Claire Shannon-Lowe, Alasdair J. Coles, Joanne L. Jones, Chris Wallace, John A. Todd, Linda S. Wicker

**Affiliations:** 1 JDRF/Wellcome Trust Diabetes and Inflammation Laboratory, Wellcome Trust Centre for Human Genetics, Nuffield Department of Medicine, National Institute for Health Research (NIHR) Oxford Biomedical Research Centre, University of Oxford, Oxford, United Kingdom.; 2 JDRF/Wellcome Trust Diabetes and Inflammation Laboratory, Wellcome Trust/MRC Building, Cambridge Institute for Medical Research, NIHR Cambridge Biomedical Research Centre, University of Cambridge, Cambridge, United Kingdom.; 3 Department of Paediatrics, MRL Wellcome Trust-MRC Institute of Metabolic Science, NIHR Cambridge Comprehensive Biomedical Research Centre, University of Cambridge, Cambridge, United Kingdom.; 4 Institute for Immunology and Immunotherapy and Centre for Human Virology, The University of Birmingham, Birmingham, United Kingdom.; 5 Department of Clinical Neurosciences, University of Cambridge, Cambridge, United Kingdom.; 6 Department of Medicine, University of Cambridge, Addenbrooke’s Hospital, Cambridge, United Kingdom, and MRC Biostatistics Unit, Cambridge Institute of Public Health, Cambridge Biomedical Campus, Cambridge, United Kingdom.

**Keywords:** Autoimmunity, Immunology, Complement, Innate immunity, T cells

## Abstract

The maintenance of peripheral naive T lymphocytes in humans is dependent on their homeostatic division, not continuing emigration from the thymus, which undergoes involution with age. However, postthymic maintenance of naive T cells is still poorly understood. Previously we reported that recent thymic emigrants (RTEs) are contained in CD31^+^CD25^−^ naive T cells as defined by their levels of signal joint T cell receptor rearrangement excision circles (sjTRECs). Here, by differential gene expression analysis followed by protein expression and functional studies, we define that the naive T cells having divided the least since thymic emigration express complement receptors (CR1 and CR2) known to bind complement C3b- and C3d-decorated microbial products and, following activation, produce IL-8 (CXCL8), a major chemoattractant for neutrophils in bacterial defense. We also observed an IL-8–producing memory T cell subpopulation coexpressing CR1 and CR2 and with a gene expression signature resembling that of RTEs. The functions of CR1 and CR2 on T cells remain to be determined, but we note that CR2 is the receptor for Epstein-Barr virus, which is a cause of T cell lymphomas and a candidate environmental factor in autoimmune disease.

## Abstract 2

Complement receptors (CR1 and CR2) and IL-8 production identify T cells that have recently left the thymus.

## Introduction

The maintenance of a diverse, naive T cell repertoire arising from the thymus (recent thymic emigrants, RTEs) is critical for health (1). Thymic involution decreases naive CD4^+^ T cell production with age in humans and is compensated for by the homeostatic maintenance of naive cells that have emigrated from the thymus earlier in life (2, 3). It was recently shown that naive T cells are retained in lymphoid tissues in a biased manner, perhaps reflecting mechanisms required for long-term retention of these cells following thymic involution (4). Naive T cells that have undergone decades of homeostatic maintenance by a low rate of division and compensatory cell death show reduced T cell receptor diversity, which has the potential to negatively impact immune defense and health (1, 5). CD31 (PECAM-1) expression identifies cells that have divided more often in the periphery (CD31^−^) from those that have not (CD31^+^), although CD31^+^ T cells still divide as a function of age as evidenced by the approximately 5-fold dilution of signal joint T cell receptor rearrangement excision circles (sjTRECs) between the ages of 20 and 60 (2, 6). This 5-fold dilution of sjTRECs equates to on average somewhat more than 2 cell divisions in the CD31^+^CD4^+^ naive T cell population over a period of 4 decades. The tyrosine-protein kinase–like 7 receptor (encoded by *PTK7*) has been reported as a marker of RTEs within the CD31^+^ naive T cell subset in adults with PTK7^+^CD31^+^ naive CD4^+^ T cells having a 3-fold enrichment of sjTRECs as compared with their PTK7^−^CD31^+^ counterparts (7). We and others have shown that CD25 is expressed at higher levels in naive CD4^+^ T cells that have divided in the periphery and that the proportions of CD31^+^CD25^+^ and CD31^−^CD25^+^ naive CD4^+^ T cells are higher with age (8, 9). CD31^+^CD25^−^ naive CD4^+^ T cells contained the highest content of sjTRECs among the 4 naive CD4^+^ T cell subsets examined: a 4-fold enrichment of sjTRECs in CD31^+^CD25^−^ naive CD4^+^ T cells as compared with their CD31^+^CD25^+^ counterparts was observed, thereby identifying the CD31^+^CD25^−^ naive CD4^+^ T cell subset as containing the highest proportion of RTEs in adults (8).

In the current study, we isolated 4 naive CD4^+^ T cell subsets from 20 adult healthy volunteers based on CD31 and CD25 expression and conducted transcriptome profiling to determine their molecular signatures. These signatures define gene expression patterns in naive T cells as they first undergo postthymic maturation and then continue to expand slowly over years and decades in the periphery waiting to encounter their cognate antigen. Unexpectedly, in the subset that has divided the least since leaving the thymus, CD31^+^CD25^−^ naive CD4^+^ T cells, complement receptor 2 (CR2) was differentially and strongly expressed as compared with both the CD31^+^CD25^+^ and CD31^−^CD25^−^ subsets. CD31^+^CD25^−^ naive CD4^+^ T cells sorted by CR2 expression showed a 6-fold enrichment of sjTRECs in the CR2^+^ fraction as compared with the CR2^−^ fraction, with the cells having the highest levels of CR2 showing the greatest enrichment (9-fold). In patients treated with a T- and B-lymphocyte-depleting drug, the anti-CD52 antibody (alemtuzumab), we observed that during homeostatic reconstitution newly emerging naive T cells from the adult thymus express high levels of CR2 and, after activation, approximately 50% produced IL-8, a chemokine (CXCL8) that can activate neutrophils and γδ T cells in microbial defense. IL-8–producing naive neonatal T cells have been recently identified (10). Although the link with RTEs was not made, this study showed enrichment of IL-8 production from CD31^+^ naive CD4^+^ T cells. Very recently, IL-8 production has been associated with RTEs in children (11). Our results also support the existence of an IL-8–producing memory T cell subset identified in a recent study (12), which may, depending on future investigations, prove to be a Th8 lineage.

## Results

### Naive CD4^+^ T cell subsets defined by CD31 and CD25 differentially express CR2 and AOAH.

**Figure 1:**
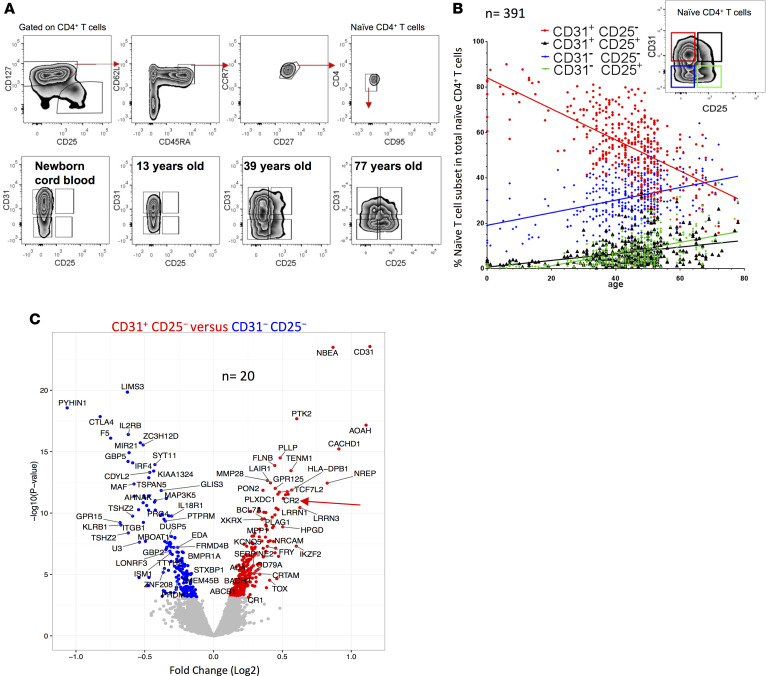
Gene expression profiling of 4 naive CD4^+^ T cell subsets identify age-related molecular signatures. (**A**) Gating strategy defining human naive CD4^+^ T cells; naive T cells were further stratified by CD31 and CD25. Representative examples (from *n* = 391; 371, 15, and 5 from cohorts 1–3, respectively; see Methods for details) of naive CD4^+^ T cells. (**B**) The proportion of naive CD4^+^ T cells as a function of age (color coding shown above graph). (**C**) Volcano plot of differences in gene expression (microarray platform) between CD31^+^CD25^−^ and CD31^–^CD25^−^ naive CD4^+^ T cells; red and blue symbols for genes with higher and lower, respectively, expression in CD31^+^CD25^−^ naive CD4^+^ T cells (*n* = 20, cohort 1).

We assessed the proportion of 4 naive CD4^+^ T cell subsets defined by CD31 and CD25 expression (8) in neonates, children, and adults in a population study of 391 donors (Figure 1, A and B). CD31^+^CD25^−^ naive CD4^+^ T cells decreased with age and this decrease was compensated for by the homeostatic maintenance of 3 subsets of naive T cells: CD31^+^CD25^+^, CD31^−^CD25^−^, and CD31^−^CD25^+^. As expected (13), the proportion of both naive CD4^+^ and CD8^+^ T cells negatively correlated with donor age (Supplemental Figure 1A; supplemental material available online with this article; https://doi.org/10.1172/jci.insight.93739DS1). To define molecules associated with the least expanded naive subset, we performed a statistically powered, genome-wide microarray RNA analysis of FACS-purified naive CD4^+^ T cells from 20 adult healthy volunteers sorted into 4 subsets based on CD31 and CD25 expression (Supplemental Figure 1B). Principal component analysis of differentially expressed genes among the 4 subsets showed a clear separation between the groups, particularly between CD31^+^ cells and their CD31^−^ counterparts (Supplemental Figure 1C). Genes with higher expression in CD31^+^CD25^−^ naive cells as compared with the CD31^−^ CD25^−^ subset included *AOAH*, which encodes the enzyme acyloxyacyl hydrolase that inactivates LPS and is highly expressed in innate immune cells such as neutrophils and DCs (14), and *CR2*, which encodes a cell surface protein that binds C3d and other complement components (15) and is also a receptor for EBV in humans (16) (Figure 1C and Supplemental Spreadsheet 1, A–D). *CR2*, *AOAH*, *TOX* (a transcription factor reported to regulate T cell development in the thymus; see ref. 17) and *CACHD1*, an uncharacterized gene that may encode a protein that regulates voltage-dependent calcium channels, were higher in CD31^+^CD25^−^ cells when they were compared with both CD31^−^CD25^−^ (Figure 1C) and CD31^+^CD25^+^ cells (Supplemental Figure 1D). A list of shared gene expression differences in naive cell subsets that have undergone more rounds of homeostatic division than CD31^+^CD25^−^ cells is shown in Supplemental Spreadsheet 1E.

Genes more highly expressed in CD31^−^CD25^−^ cells as compared with CD31^+^CD25^−^ cells (Figure 1C) are consistent with the occurrence of activation and differentiation events during the homeostatic maintenance of naive T cells. The genes include *PYHIN1*, encoding an interferon-induced intracellular DNA receptor that is a member of a family of proteins that induces inflammasome assembly (18, 19); *GBP5*, encoding a protein that promotes NLRP3 inflammasome assembly (20); *CTLA4*, a negative regulator of T cell activation; *KLRB1*, encoding CD161, a marker of IL-17–producing T cells (21); and *IL2RB*, a signaling subunit of the trimeric high-affinity IL-2 receptor.

### Naive T cells expressing CR2 are more prevalent in children than adults.

**Figure 2:**
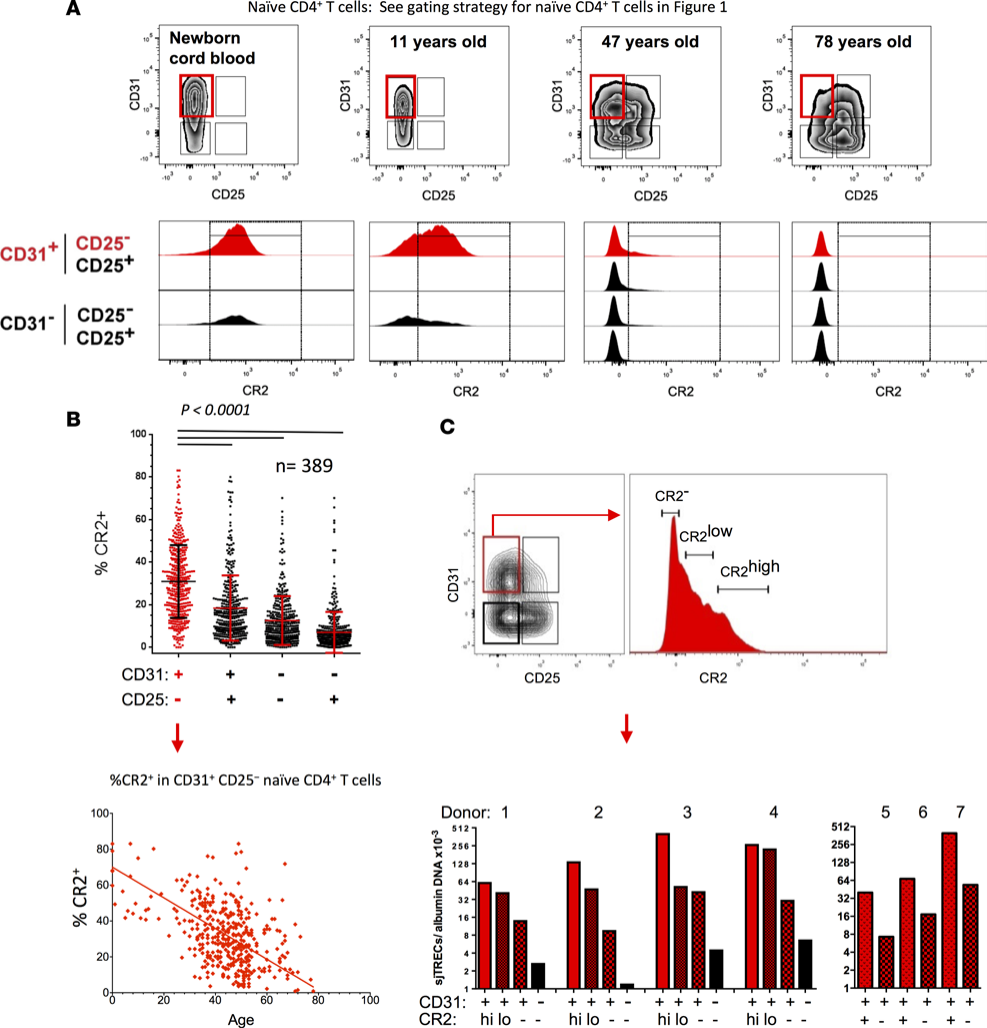
CR2 marks the most naive CD4^+^ T cell subset. (**A**) Representative examples of CR2 expression in naive T cell subsets. (**B**) Percentage CR2^+^ cells in each subset and frequency of CR2^+^ cells in the CD31^+^CD25^−^ naive CD4^+^ T cell subset as a function of age (from *n* = 389; 371, 15, and 3 from cohorts 1–3, respectively). Significance determined by paired *t* test. (**C**) Representative sorting strategy for CD31^+^CD25^−^ naive CD4^+^ T cells identified as CR2^−^, CR2^lo^, and CR2^hi^ (donors 1–4). For donors 5–7, the CR2^+^ gate is a combination of low- and high-CR2-expressing cells. Sorted cells were assessed for signal joint T cell receptor rearrangement excision circles (sjTRECs) (*n* = 7; 1 and 6 donors from cohorts 1 and 3, respectively).

We verified the CR2 mRNA expression results using flow cytometric analysis, with the CD31^+^CD25^−^ naive CD4^+^ T cell subset from adults and children having the highest proportion of cells positive for CR2 (Figure 2, A and B). In the case of cord blood, greater than 60% of CD31^−^ naive cells also expressed CR2. The 3 subsets previously shown to be products of homeostatic turnover based on sjTREC content (8) had lower proportions of CR2^+^ cells, with the percentage CR2^+^ decreasing from CD31^+^CD25^+^ cells to CD31^−^CD25^−^ to CD31^−^CD25^+^. The lower percentages of CR2^+^ cells within the CD31^+^CD25^−^ naive subset by age (Figure 2B) was more pronounced when considered out of total CD4^+^ T cells since the proportion of naive cells within the CD4^+^ population decreases with age (Supplemental Figure 2A). The CR2^+^ fraction of the CD31^+^CD25^−^ naive CD4^+^ T cell subset also had the highest level of CR2 on a per-cell basis among the 4 naive subsets in a given adult or child and this density decreased with age (Figure 2A). Cord blood was again the exception where CD31^+^ and CD31^−^ cells had similarly high CR2 levels. A pattern similar to that of naive CD4^+^ T cells from adults and children of fewer CR2^+^ cells and a lower density of CR2 per cell with age was also observed on naive CD8^+^ T cells (Supplemental Figure 2B).

Although CR2 expression on CD31^+^CD25^−^ naive CD4^+^ T cells in adults varies greatly, this most likely reflects the biological variation of thymic output and rate of homeostatic division. Supporting the hypothesis that CR2 expression on human naive T cells is influenced by time in the periphery, we observed that the percentage of CD31^+^CD25^−^ naive CD4^+^ T cells that are CR2^+^ was stable in 10 donors during a period of time in which little homeostatic division would have occurred (second sample taken 11 to 17 months after the first) (Supplemental Figure 2C). The regulation of CR2 in naive T cells is distinct from that in B cells where CR2 expression is observed on the majority of both mature naive and memory B cells (22) and expression levels on CR2^+^ B cells are approximately 30-fold higher than those on CR2^+^ naive T cells (Supplemental Figure 2D). Indeed, to optimize detection of CR2 on naive T cells we stained simultaneously with 2 anti-CR2 antibody clones. Activation of B cells has been shown to increase CR2 promoter activity and CR2 protein levels (23), whereas CR2 mRNA decreases in naive T cells following anti–CD3/CD28 activation (Supplemental Spreadsheet 3), suggestive of distinct mechanisms of regulation in these 2 lymphocyte subsets. Because PTK7 has been described as a marker of RTE (7, 11), we examined our microarray gene expression data for differential expression in the 4 subsets of naive cells in adults to determine if a pattern similar to that observed for *CR2* could be detected. Although no differential expression was evident in any of the comparisons (Supplemental Spreadsheet 1, A–D), this appears to be due to the fact that the levels of *PTK7* mRNA were not above background, consistent with the very low levels of PTK7 mRNA and protein expression previously reported in adult naive CD4^+^ T cells (see Figure 2 in ref. 7).

### CR2^+^ naive CD4^+^ T cells have a higher sjTREC content than their CR2^−^ counterparts.

To determine whether CR2 is a molecular marker of the subset of CD31^+^CD25^−^ naive CD4^+^ T cells that have divided the least in the periphery since emigrating from the thymus, we sorted CR2^hi^, CR2^lo^, and CR2^−^CD31^+^CD25^−^ naive CD4^+^ T cells along with CD31^−^CD25^−^ naive CD4^+^ T cells from 4 adult donors and, when cell numbers were limiting, CR2^+^ and CR2^−^CD31^+^CD25^−^ naive CD4^+^ T cells from 3 additional adult donors, and assessed sjTREC levels (Figure 2C). Purified CD31^−^ naive CD4^+^ T cells had fewer sjTRECs than any of the CD31^+^ populations (Figure 2C) as expected based on previous studies showing that the loss of CD31 expression from naive cells is associated with a substantial loss of sjTRECs (2, 6, 8). CD31^+^CR2^−^ and CD31^+^CR2^hi^ cells had on average 7- and 67-fold enrichment, respectively, of sjTRECs as compared with CD31^−^CR2^−^ cells. CR2^+^ cells had more sjTRECs than CR2^−^ cells in all cases (*n* = 7, *P* = 0.0023 using sjTREC values obtained from CR2^hi^CD31^+^ cells compared with CR2^−^CD31^+^ cells for donors 1–4 and CR2^+^CD31^+^ cells compared with CR2^−^CD31^+^ cells for donors 5–7; *P* = 0.018 using CR2^lo^CD31^+^ cells compared with CR2^−^CD31^+^ cells for donors 1–4 and CR2^+^CD31^+^ cells compared with CR2^−^CD31^+^ cells for donors 5–7, Mann-Whitney rank test). This result indicates that in adults CR2^+^ cells have undergone fewer rounds of homeostatic division as compared with CR2^−^ cells within the CD31^+^CD25^−^ subset of naive CD4^+^ cells. Enrichment of sjTRECs was 9.2-fold comparing CR2^hi^ and CR2^−^CD31^+^ cells in donors 1–4 and 5.6-fold when comparing CR2^+^ and CR2^−^CD31^+^ cells in donors 5–7. Where cell numbers were sufficient to separate the CR2^hi^ and CR2^lo^ naive CD4^+^ T cells, CR2^lo^ cells had fewer sjTRECs than the CR2^hi^ cells in each comparison (*n* = 4, *P* = 0.11), suggesting that higher CR2 expression on a per-cell basis on CD31^+^CD25^−^ naive CD4^+^ T cells identifies cells that have divided the least number of times since leaving the thymus. These observations along with our previous demonstration of CD25 expression on homeostatically expanded naive CD31^+^ cells T cells (8) explain why naive CD4^+^ T cells isolated only by CD31 expression show an age-dependent loss of sjTRECs (2, 6).

### RTEs from the adult thymus are CR2^+^.

**Figure 3:**
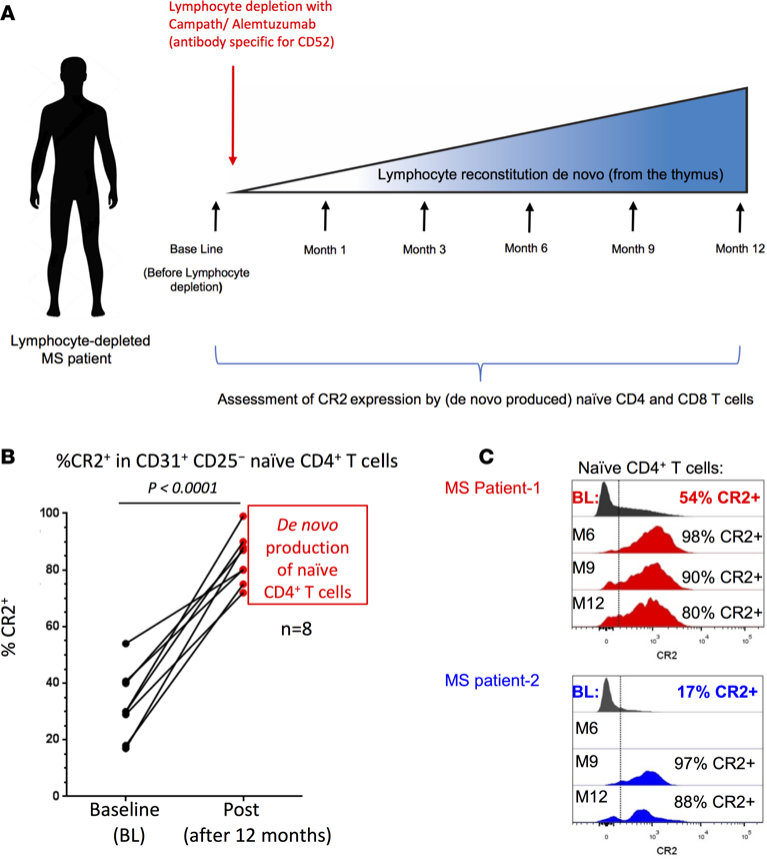
Higher complement receptor 2 (CR2) expression by human naive CD4^+^ T cells during de novo reconstitution. (**A**) Treatment and sampling time points of multiple sclerosis (MS) patients. (**B**) Frequency of CD31^+^CD25^−^ naive CD4^+^ T cells expressing CR2 in MS patients before (baseline, BL) and 12 months after lymphocyte depletion with anti-CD52 (Campath). Significance determined by paired *t* test. (**C**) CR2 expression on CD31^+^CD25^−^ naive CD4^+^ T cells from 2 patients before and at various times during reconstitution; time points from 6 additional patients are shown in Supplemental Figure 3C.

To further test the hypothesis that CR2 expression on naive T cells defines RTEs throughout life rather than being specific to cells generated during the neonatal period, we monitored newly generated naive CD4^+^ T cells in 8 multiple sclerosis (MS) patients depleted of T and B cells using alemtuzumab (24) (Figure 3A and Supplemental Figure 3A). Twelve months after depletion, in all 8 patients, the proportion of naive CD4^+^ (Figure 3, B and C, and Supplemental Figure 3C) and CD8^+^ (Supplemental Figure 3B) T cells expressing CR2 was increased as compared with baseline. These data demonstrate that de novo RTEs produced in the adult thymus can also be stratified by CR2 expression, similar to our findings based on CR2 expression on naive T cells in cord blood and in the peripheral blood of children (Figure 2, A and B). Interim time points were available from most patients (Figure 3C and Supplemental Figure 3C), showing that when the first few naive CD4^+^ T cells were detected after depletion (3–9 months after treatment), they were essentially all CR2^+^ with a density of CR2 per cell equalling that seen in cord blood (Figure 2A). This observation was independent of whether a patient had good or poor naive CD4^+^ T cell reconstitution overall.

A potential utility of our observation is to use CR2 as a biomarker of thymic reserve. Therefore, we compared the frequency of CR2^+^ cells within the CD31^+^CD25^−^ naive CD4^+^ T cell subset prior to lymphocyte depletion with the ability of the thymus to reconstitute naive CD4^+^ T cells (Supplemental Figure 3A). The 2 patients (P2 and P6) with the lowest level of naive CD4^+^ T cell reconstitution at 12 months (5.4% and 2.4% of baseline, quantified as number of cells per ml of blood) had the lowest levels of CR2^+^ T cells within their CD31^+^CD25^−^ naive CD4^+^ T cell subset (18% and 17%) prior to treatment. In contrast, the 6 patients who reconstituted their naive CD4^+^ T cell pool from 13% to 64% of baseline (average of 37%) by 12 months had on average 38% (range 29%–54%) CR2^+^ cells in the CD31^+^CD25^−^ naive CD4^+^ T cell subset prior to treatment. Although these results are encouraging for the use of CR2 as a biomarker of T cell production by the thymus, additional patients will need to be examined to verify our findings.

### Gene expression profiling of CR2^+^ and CR2^−^ naive cells.

**Figure 4:**
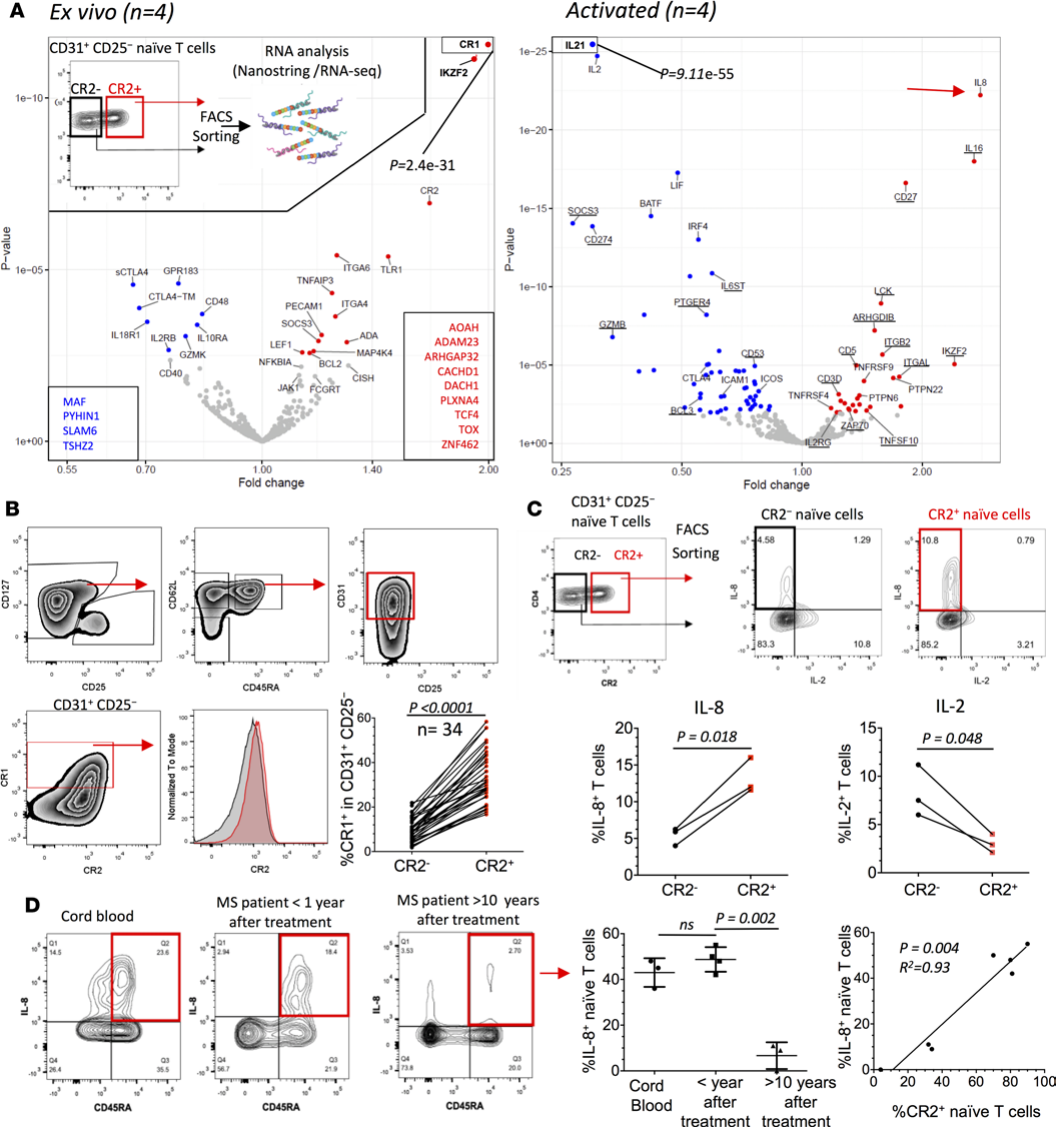
CR2^+^ naive CD4^+^ T cells have a unique molecular signature. (**A**) Volcano plots of differences in gene expression (NanoString platform) between CR2^+^ versus CR2^−^ naive CD4^+^ T cells (gating strategy shown as insert) ex vivo and after activation (anti–CD3/CD28). Genes expressed at a higher or lower level in CR2^+^ cells have red or blue symbols, respectively. Underlined genes have lower expression after activation. Genes in boxes are from the RNA-seq platform (*n* = 4 adult donors from cohorts 1 and 3). (**B**) Ex vivo CR1 protein expression on CR2^+^ and CR2^−^ cells (*n* = 34, age range 0–67, cohorts 1–3, paired *t* test). Red and gray histograms gated on CR1^+^ and CR1^−^ cells, respectively. (**C**) Representative histograms and compiled frequencies of cytokine production following activation of CR2^+^ and CR2^−^ cells sorted from CD31^+^CD25^−^ naive CD4^+^ T cells (*n* = 3, age range 30–44, cohort 3, paired *t* test). (**D**) Representative histograms of IL-8 production from isolated CD4^+^ T cells following activation with PMA and ionomycin. Compiled data of percentage IL-8^+^ cells out of naive (CD45RA^+^) CD4^+^ T cells (unpaired *t* test, *n* = 3 cord bloods from cohort 3, 4 multiple sclerosis [MS] patients 6 to 9 months after treatment, 3 MS patients >10 years after treatment. Correlation of percentage IL-8^+^ cells (following activation) and percentage CR2^+^ cells (assessed prior to activation) in the MS patients (*n* = 7).

To evaluate the potential function of CR2^+^ naive CD4^+^ T cells we compared RNA isolated from sorted CR2^+^ and CR2^−^CD31^+^CD25^−^ naive CD4^+^ T cells ex vivo and after activation with anti–CD3/CD28 (Figure 4A, Supplemental Spreadsheets 2 and 3, and Supplemental Tables 1 and 2). This analysis revealed a unique transcriptional signature of the CR2^+^ RTEs. Complement receptor 1 (*CR1*), *AOAH*, and *TLR1* were more highly expressed in CR2^+^ cells. Coexpression of CR1 and CR2 was observed on the surface of CD31^+^CD25^−^ naive CD4^+^ T cells from healthy controls (Figure 4B) and MS patients reconstituting their T cells after alemtuzumab treatment (Supplemental Figure 4A). Coexpression of CR1 and CR2 was also observed on naive CD8^+^ T cells (Supplemental Figure 4, B and C). Consistent with CR2 marking RTEs, we observed that PTK7 mRNA was approximately 2-fold more abundant in the CR2^+^ as compared with the CR2^−^CD31^+^CD25^−^ naive CD4^+^ T cells. In comparison, CR2 mRNA read counts were 10-fold more abundant in CR2^+^ as compared with the CR2^−^ cells and there were 20-fold fewer PTK7 mRNA reads as compared with CR2 mRNA reads (Supplemental Figure 4D and Supplemental Spreadsheet 2). Since both CR2 and PTK7 mRNA levels appear to correspond to protein levels in naive CD4^+^ T cells (Supplemental Table 1 in this study and ref. 7, respectively), the RNA sequencing (RNA-seq) data support the previous observation that PTK7 levels are very low on adult naive T cells.

Following activation, IL-8 mRNA was more highly expressed in CR2^+^ naive CD4^+^ T cells whereas *IL2*, *IL21*, *LIF*, and *IFNG* were more highly expressed in CR2^−^ cells (Figure 4A, Supplemental Spreadsheet 3, and Supplemental Table 2). This is consistent with studies showing that RTEs from children secrete less IFN-γ and IL-2 (7) and more IL-8 (11). *TNF*, *LTA*, and *IL23A* were highly upregulated with activation but there was no difference between the CR2^+^ and CR2^−^ naive subsets. IL-8 mRNA upregulation was of particular interest since it has been identified as a phenotype of neonatal naive cells (10). We therefore measured IL-8 and IL-2 protein production from sorted CR2^+^ versus CR2^−^ naive CD4^+^ T cells after activation with PMA and ionomycin. We verified the RNA results showing that IL-8 is preferentially produced by the CR2^+^ subset, whereas the opposite is the case for IL-2 (Figure 4C). The measurement of percentage positive for IL-8 is an underestimate of the difference between CR2^+^ versus CR2^−^ naive CD4^+^ T cells, since for the CR2^+^ cells expressing IL-8 the production of IL-8 on a per-cell basis was greater than for CR2^−^ cells (mean fluorescence intensity 7,000 ± 177 [SEM] vs. 4,875 ± 403 [SEM], *P* = 0.008, unpaired *t* test). Because CR2 rapidly disappears from the surface of T cells activated in vitro when PMA and ionomycin are used to induce cytokine expression (Supplemental Figure 4E), analysis of cytokine production after stimulation is prevented unless cells are sorted by CR2 expression first as in Figure 4C. Therefore, for other samples, namely isolated CD4^+^ T cells from cord blood and from peripheral blood of MS patients reconstituting their naive T cell compartment and MS patients in whom naive T cells had undergone over a decade of homeostatic division, we correlated IL-8 production in naive CD4^+^ T cells with the frequency of CR2^+^ cells within the same naive T cell subset prior to activation (Figure 4D). Notably, all MS patients that were less than 1 year after depletion (*n* = 4) had CR2 expression on 70% or more of their naive CD4^+^ T cells (similar to the patients described in Figure 3 and Supplemental Figure 3). Approximately 50% of these naive CD4^+^ T cells produced IL-8. IL-8 production by naive cells from these patients was as prevalent as that observed from cord blood naive CD4^+^ T cells (*n* = 3). These data support the hypothesis that RTEs in adults recapitulate the developmental stage observed in neonatal RTEs. On the other hand, the frequency of naive CD4^+^ T cells expressing CR2 and CD45RA^+^CD4^+^ T cells producing IL-8 in MS patients more than 10 years after depletion (*n* = 3) was less than 35% and 12%, respectively. A correlation between CR2 expression and IL-8 production was therefore observed in the 7 MS patients examined. Where homeostatic division had occurred for at least a decade (Figure 4D), MS patients were similar to healthy adults (Figure 4C). Since the expression of CR2 on a per-cell basis is lower in adults than in neonates and children (Figure 2A), and the number of sjTRECs is fewer in cells with lower CR2 expression on a per-cell basis (Figure 2C), these data suggest that as naive cells divide homeostatically, CR2 expression decreases as well as their ability to produce IL-8.

### CR2^+^ central memory cells produce IL-8.

**Figure 5:**
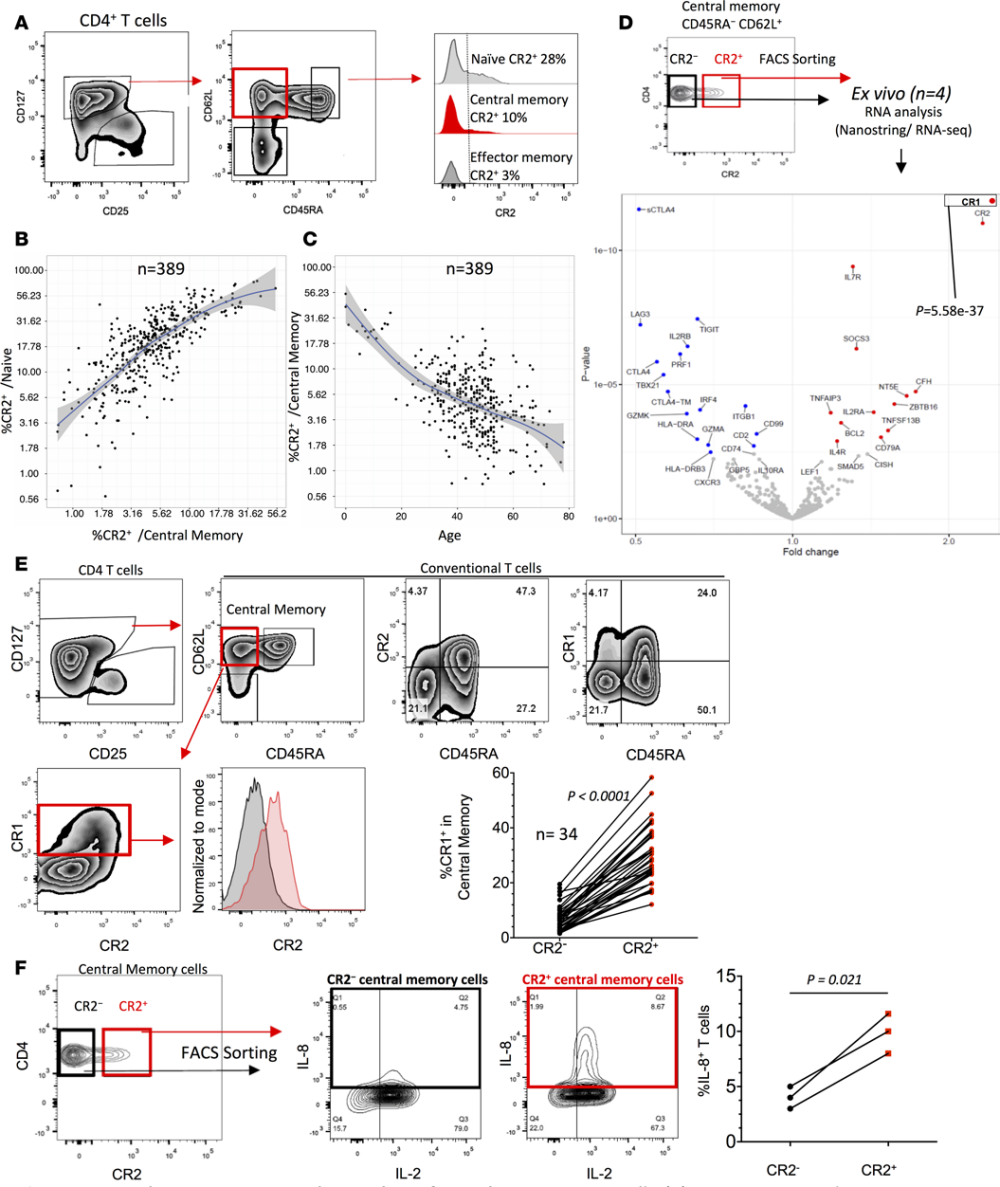
CR2 and CR1 are coexpressed on a subset of central memory CD4^+^ T cells. (**A**) Gating strategy and CR2 expression. Correlation of CR2 expression on central memory versus naive CD4^+^ T cells (**B**) and CR2^+^ central memory CD4^+^ T cells versus age (**C**) (*n* = 389; 371, 15, and 3 from cohorts 1–3, respectively). (**D**) Gating example of central memory cells sorted by CR2 expression, and gene expression analysis (NanoString). Color coding is described in Figure 4. (**E**) FACS analysis and compiled data of CR1 and CR2 coexpression (*n* = 34, age range 0–67, cohorts 1–3, paired *t* test). Red and gray histograms gated on CR1^+^ and CR1^−^ cells, respectively. (**F**) Example and compiled data of IL-8 production from sorted and activated CR2^+^ and CR2^−^ memory CD4^+^ T cells (*n* = 3, age range 30–44, cohort 3, paired *t* test).

When analyzing IL-8 production by CD4^+^ T cells we noted that in MS patients more than 10 years past lymphocyte depletion, a small fraction of activated CD45RA^−^ memory CD4^+^ T cells produced IL-8 (Figure 4D). We therefore hypothesized that CD4^+^ memory T cells can be expanded from IL-8–producing CR2^+^ naive T cells in vivo. CR2 expression was observed on a proportion of central and effector memory CD4^+^ T cells, and Tregs expressed the lowest levels of CR2 (Figure 5A and Supplemental Figure 5A). CR2 expression on memory cells correlated with CR2 expression by naive T cells (Figure 5B) and was age dependent (Figure 5C), suggesting that with age the CR2^+^ central memory cells either lose CR2 expression or the subset contracts due to competition with CR2^−^ central memory cells. CR2^+^ central memory CD8^+^ T cells were also observed and their frequency was similarly age dependent (Supplemental Figure 5B). As seen with the equivalent CD4^+^ naive T cell subsets, RNA analysis of sorted CR2^+^ and CR2^−^ central memory CD4^+^ T cells showed CR1 to be the most differentially expressed gene (Figure 5D, Supplemental Spreadsheet 4, and Supplemental Table 1), a phenotype confirmed at the protein level (Figure 5E). An overall expression analysis of the CR2^+^ and CR2^−^ naive and central memory cell subsets confirmed that the 2 memory populations clustered together away from the 2 naive subsets, confirming that the CR2^+^ central memory cells are bona fide central memory cells, not an unusual naive cell subset (Supplemental Figure 5C). Sorted CR2^+^ central memory cells produced higher levels of IL-8 after activation as compared with CR2^−^ central memory cells, and unlike naive CR2^+^ cells (Figure 4C), all central memory cells producing IL-8 also produced IL-2 (Figure 5F).

Among the differentially expressed genes between the CR2^−^ and CR2^+^ central memory cells, a gene of particular note is complement factor H (*CFH*), which is upregulated in both CR2^−^ and CR2^+^ central memory cells as compared with naive cells but has 2.7-fold higher levels in CR2^+^ central memory cells compared with CR2^−^ central memory cells (Figure 5D, Supplemental Spreadsheet 4, and Supplemental Table 1). Genes with shared expression differences between CR2^+^ naive and CR2^+^ central memory T cells versus their CR2^−^ counterparts ex vivo are highlighted in Supplemental Figure 6 and include a potentially relevant transcription factor for CR2^+^ CD4^+^ cells, *ZNF462*, which is expressed 13.0-fold higher in CR2^+^ versus CR2^−^ naive cells and 7.2-fold higher in CR2^+^ versus CR2^−^ central memory cells. Four genes, *ADAM23*, *ARHGAP32*, *DST*, and *PLXNA4*, shared by the 2 CR2^+^ subsets may enhance migratory properties that augment host surveillance (25).

## Discussion

An understanding of the impact of thymic involution on health remains incomplete despite its influence on the aging of the immune system (1) and other clinical aspects (24, 26–28). In our analysis of naive T cell subsets, we report for the first time to our knowledge coexpression of CR1 and CR2 on the most naive T cells and that these, as well as other molecules differentially expressed by these cells, will enable a better understanding of human RTEs.

Coexpression of CR1 and CR2 occurs on follicular DCs and B cells and their functions are critical to generating antibody responses, including the retention of immune complexes on follicular DCs (29, 30). CR1 participates in the degradation of activated C3 to C3d, which then binds CR2 and facilitates the interaction via immune complexes of follicular DCs with B cells, lowering their threshold of activation. The presence of CR2 and CR1 on the surface of naive T cells provides them with the potential to participate in immune responses in a manner analogous to follicular DCs and B cells. Our findings add to an increasing appreciation of the role in T cell functions of molecules classically described as belonging to the innate immune system. Previously, it has been shown that human T cells express and process C3 to C3a and C3b and binding of C3b by CD46 regulates cytokine production and effector differentiation (31, 32). There have been previous reports of CR2 expression on fetal T cells (33) and thymocytes (34), but not in the context of RTEs as presented here. Evidence for a CR2 and CR1 function in T cells was their 3- to 10-fold enhancement of C3-dependent HIV infection in the HPB-ALL T cell leukemia cell line (35).

The hypothesis that RTEs have a distinctive ability to respond to bacterial pathogens as compared with long-term peripheral naive T cells was supported by our finding that following activation CR2^+^CD31^+^CD25^−^ RTEs preferentially produced IL-8 (CXCL8). The IL-8 chemokine has been recently characterized as a “proinflammatory immunoprotective cytokine of neonatal T cells” via neutrophil recruitment and costimulation of γδ T cells (10). The authors also demonstrated that IL-8 expression by neonatal T cells was increased when in addition to TCR stimulation cells were provided with bacterial flagellin or the TLR1/2 agonist Pam3Cys. In our study, not only have we observed that approximately 50% of naive CD4^+^ T cells in cord blood produce IL-8 upon activation, we also demonstrated a similar proportion of IL-8–producing naive T cells in the blood of MS patients, with newly generated naive T cells appearing following T and B cell depletion with alemtuzumab (Figure 3 and Supplemental Figure 3). In healthy adults or in MS patients greater than 10 years following lymphocyte depletion, a smaller portion of naive CD4^+^ cells produced IL-8, but we also noted that the level of CR2 on a per-cell basis is much lower on CR2^+^ naive T cells from adults as compared with the level of CR2 expression observed on CR2^+^ naive T cells present in neonates, children, and adults actively reconstituting their immune system. This is consistent with the observation that in adult naive CD4^+^ T cells sorted by CR2 levels, the greatest number of sjTRECs was in cells with the highest CR2 levels per cell (9-fold enrichment of sjTRECs in the CR2^hi^ fraction of CD31^+^CD25^−^ naive CD4^+^ T cells as compared with the CR2^−^ fraction in the same subset). Overall, we conclude that as naive cells emigrate from the thymus they express high levels of CR2 and are capable of secreting IL-8, but as homeostatic division of naive T cells occurs through time, CR2 levels and the preferential secretion of IL-8 declines. Our findings are compatible with recent studies showing that following neonatal thymectomy IL-8 production by naive CD4^+^ T cells was reduced by greater than 90% (11). Taken together, recent development in the thymus, not absolute age, confers a unique phenotype to RTEs: preferential IL-8 production and high CR2 expression. We also noted at the mRNA level higher expression in RTEs of the genes encoding TLR1, a bacterial pattern recognition receptor, and AOAH, a secreted enzyme that inactivates LPS.

We hypothesize that following their emigration from the thymus, T cells initially express high levels of CR2 and CR1, secrete IL-8 and TNF, have the capacity to hydrolyze LPS, based on mRNA results, and produce lower levels of T cell cytokines such as IL-2 and IFN-γ. It is possible that this differentiation state provides tissue protection while avoiding overwhelming T cell activation, attributes likely to be critical to the newborn encountering a wide range of antigens from microbes on the skin and mucosal tissues as well as encountering airborne and food antigens. Our results are consistent with previous mouse and human studies demonstrating decreased IL-2 and INF-γ production by activated RTEs compared with T cells resident in the periphery for a longer period of time (11, 36, 37). Studies focused on the biology of RTEs in mice demonstrated that in the absence of inflammation RTEs display heightened susceptibility to tolerance induction to tissue-restricted antigens (36), suggesting that the RTE differentiation state could also contribute to tolerance induction to commensal bacteria. Such tolerance induction may require RTE migration into all tissues and associated draining lymph nodes that are exposed to commensals as well as pathogens. The importance of tissue residency by naive T cells is strongly supported by recent discoveries showing that pediatric samples of colon, ileum, jejunum, and lung, in contrast to tissues from young adults, contain a large portion of naive CD4^+^ and CD8^+^ T cells, most of which were defined as RTEs by the virtue of expressing CD31 (37). Results from TCR repertoire analyses of naive T cells isolated from lymphoid tissues of donors aged 2 months to 73 years suggest naive T cell homeostatic division is at least in part site-specific and that the dynamics of naive T cell recirculation in humans may differ from those understood from mouse studies (4).

Aspects of the innate signature of RTEs are retained by a subset of CR2^+^ central memory T cells that express CR1 and secrete IL-8 upon activation, suggesting that RTEs are precursors of a subset of memory T cells (see genes shared by CR2^+^ naive and memory T cells in Supplemental Figure 6). Further supporting this hypothesis, we noted that when compared with their CR2^−^ counterparts, CR2^+^ central memory T cells express 3-fold higher *CFH*, a complement regulatory glycoprotein possessing a cofactor activity for complement inactivation on the surface of the host cells, but not on that of the pathogen (15). However, since we did not observe differential expression of the genes encoding Th-defining transcription factors (38) — *RORC* (Th17), *GATA3* (Th2), *PRDM1* (Tfh), and *BCL6* (Tfh) (although differential expression of *TBX21* [Th1] was observed, Supplemental Spreadsheet 4) — when comparing CR2^+^ and CR2^−^ memory T cells, this implies that CR2^+^ naive T cells can differentiate to Th memory lineages not characterized by IL-8 secretion while maintaining CR2 expression. Wong and colleagues (12) noted the possible developmental relationship of IL-8–producing memory T cells in tissues with the abundant IL-8–secreting naive T cells in cord blood and noted that “while not defined as a Th lineage, IL-8–producing cells had very little overlap with other Th subsets in terms of cytokine secretion and trafficking receptor expression.” We suggest that the shared gene expression pattern of memory and naive CR2^+^ cells that preferentially secrete IL-8, including the transcription factor ZNF462, support a developmental relationship.

Future functional studies of CR2 and CR1 on naive and memory CD4^+^ and CD8^+^ T cells will further our understanding of T cell biology, thymic function as people age and during bone marrow transplantation (28), HIV infection (26), thymectomy (7, 11), and immune reconstitution following immune depletion (24) or chemotherapy (27). Recently, the functions of 2 other complement receptors expressed in T cells, C5aR1 and C5aR2, have been described. C5 activation in T cells was shown to be required for NLRP3 inflammasome assembly and Th1 differentiation (39). The presence of EBV receptors (CR2 molecules) on T cells highlights a potential pathway of EBV infection that in some cases results in T cell lymphoma (40). It is possible that the binding of EBV via CR2 to EBV-specific, CR2^+^ naive T cells during antigen-specific activation beneficially modulates responsiveness, thereby accounting for the observation that the severity of EBV increases with age (41), which correlates with the loss of CR2 on naive T cells with homeostatic division. The near absence of CR2^+^ T cells later in life could contribute to the less effective immunity, especially to microbial infections, observed in older individuals (3).

## Methods

### Human samples.

Donors of peripheral blood volunteered for 1 of 3 observational studies (cohorts 1–3 below) and 1 clinical trial. The 3 cohorts are described below and how each cohort contributed to the results presented in the manuscript. Although cohorts 1–3 involved projects with research questions concerning type 1 diabetes and had type 1 diabetic participants, none of the donors included in this study had type 1 diabetes. Aside from 4 of the 371 donors in cohort 1 forming a majority of the immunophenotyping panel (detailed below), all participants in cohorts 1–3 self-reported as healthy.

Cohort 1 consisted of donors participating in the following study: Genes and Mechanisms in Type 1 Diabetes in the Cambridge BioResource recruited via the National Institute for Health Research (NIHR) Cambridge BioResource. Within cohort 1 are a group of 371 donors who were immunophenotyped for Figure 1B, Supplemental Figure 1A, Figure 2B, Supplemental Figure 2, A and B, Figure 5, B and C, and Supplemental Figure 5, A and B (114 males aged 19 to 72, median 47; 257 females aged 18 to 78, median 46; all self-reported as healthy except for 4 female donors who self-reported a history of autoimmune disease — 2 with autoimmune thyroid disease, 1 with vitiligo, and 1 with celiac disease). This cohort also includes an additional 20 donors (aged 18–65, 7 males and 13 females) who donated samples for the microarray analyses reported in Figure 1C, Supplemental Figure 5, C and D, and Supplemental Spreadsheet 1. An additional 3 donors (2 females, 40–44 years old, 1 female, 30–34 years old) provided samples for the RNA expression studies (NanoString and RNA-seq, Figure 4A, Figure 5D, Supplemental Tables 1 and 2, and Supplemental Spreadsheets 2–4). One donor (female, 40–44 years old) provided samples for sjTREC determinations shown in Figure 2C. Finally, 14 additional donors (age 26–67, 5 males and 9 females) from this study were assessed for CR1 levels in Figure 4B, Supplemental Figure 4B, and Figure 5E. The study was approved by NRES Committee East of England - Norfolk (ref: 05/Q0106/20).

Cohort 2 consisted of donors participating in the following study: Diabetes—Genes, Autoimmunity, and Prevention, a study of newly diagnosed children with type 1 diabetes and nondiabetic siblings of probands with type 1 diabetes. All 15 donors (7 males aged 5–16, 8 females aged 1–14) did not have diabetes and were negative for type 1 diabetes–related autoantibodies). These 15 donors contributed to the immunophenotyping presented in Figure 1B, Supplemental Figure 1A, Figure 2B, Supplemental Figure 2, A and B, Figure 5, B and C, Supplemental Figure 5, A and B, Figure 4B, Supplemental Figure 4B, and Figure 5E.

Cohort 3 consisted of donors participating in the following study: Investigating Genes and Phenotypes Associated with Type 1 Diabetes (8 cord blood samples; 7 adults, none with self-reported autoimmunity). Six adult donors (3 males and 3 females, 18–45 years of age) provided samples for sjTREC determinations shown in Figure 2C. One donor (female, 35–39) provided a sample for the RNA expression studies (NanoString and RNA-seq, Figure 4A, Figure 5D, Supplemental Tables 1 and 2, and Supplemental Spreadsheets 2–4). Three adult donors (1 male and 2 females, 30–44 years of age) provided samples for IL-8 and IL-2 protein production following activation in Figure 4C and Figure 5F. Three cord blood samples were utilized to assess IL-8 protein production in Figure 4D and were used for immunophenotyping in Figure 1B, Supplemental Figure 1A, Figure 2B, Supplemental Figure 2, A and B, Figure 5, B and C, Supplemental Figure 5, A and B, Figure 4B, Supplemental Figure 4B, and Figure 5E. Immunophenotyping in Figure 1B, Figure 4B, Supplemental Figure 4B, and Figure 5E included 2 additional cord bloods.

The MS patients (6 females and 2 males, aged 27–49) studied longitudinally (Figure 3 and Supplemental Figure 3) were from the placebo arm (received alemtuzumab only, keratinocyte growth factor was not given) of the trial “Keratinocyte Growth Factor - promoting thymic reconstitution and preventing autoimmunity after alemtuzumab (Campath-1H) treatment of multiple sclerosis” (REC reference: 12/LO/0393, EudraCT number: 2011-005606-30). An additional 11 adult MS patients studied in a cross-sectional manner were treated with alemtuzumab 6 to 9 months (*n* = 4, Figure 4D), 6 to 12 months (*n* = 4, Supplemental Figure 3, A and C), or greater than 10 years (*n* = 3, Figure 4D) prior to sample donation (CAMSAFE REC 11/33/0007).

Diabetes—Genes, Autoimmunity, and Prevention was originally approved by the National Research Ethics Committee London – Hampstead, and is now held under the ethics of Investigating Genes and Phenotypes Associated with Type 1 Diabetes, which was approved by NRES Committee East of England - Cambridge Central (ref: 08/H0308/153). NIHR Cambridge BioResource donors were collected with the prior approval of the National Health Service Cambridgeshire Research Ethics Committee. DBD, LSW, and JAT codesigned the Diabetes—Genes, Autoimmunity, and Prevention study. FWL helped organize the provision of the blood samples in the Investigating Genes and Phenotypes Associated with Type 1 Diabetes study. JAT led the Investigating Genes and Phenotypes Associated with Type 1 Diabetes and Genes and Mechanisms of Type 1 Diabetes in the Cambridge BioResource studies.

### Study approval.

Written informed consent was obtained from all participants or their guardians taking part in the study.

### Whole blood and PBMC immunostaining.

Blood samples were directly immunophenotyped within 5 hours following donation. Samples were blocked for 10 minutes with mouse IgG (20 μg/ml), stained for 40 minutes at room temperature with appropriate antibodies, and then lysed with freshly prepared 1× BD FACS Lysing Solution (BD Biosciences). After lysis of red blood cells, samples were washed with BD CellWASH (BD Biosciences). Finally, the samples were fixed with freshly prepared 1× BD CellFIX (BD Biosciences). The samples were stored at 4°C in the dark until analysis using a BD LSRFortessa flow cytometer. Peripheral blood mononuclear cell (PBMC) samples, prepared as previously described (42), were blocked for 10 minutes, stained for 1 hour at 4°C, washed twice, and fixed as described for peripheral blood immunophenotyping except for intracellular staining when surface-stained cells after the wash step were placed in FOXP3 Fix/Perm buffer (eBioscience). Phenotyping panels are detailed in Supplemental Table 3. CD25 detection sensitivity was increased (42) by simultaneous application of 2 anti-CD25 monoclonal antibodies labeled with the same fluorochrome (clones 2A3 and M-A251, BD Biosciences). Antibody concentrations used were based on the manufacturer’s instructions as well as on optimization studies. Appropriate isotype controls and fluorescence-minus-one conditions were used during the development of staining panels. Immunostained samples were analyzed on a BD LSRFortessa cell analyzer and data were visualized using FlowJo (Tree Star).

### Cryopreserved PBMCs.

PBMC isolation, cryopreservation, and thawing were performed as previously described (42). In brief, PBMC isolation was carried out using Lympholyte (CEDARLANE). PBMCs were cryopreserved in heat-inactivated, filtered human AB serum (Sigma-Aldrich) and 10% DMSO (Hybri-MAX, Sigma-Aldrich) at a final concentration of 10 × 10^6^/ml and were stored in liquid nitrogen. Cells were thawed in a 37°C water bath for 2 minutes. PBMCs were subsequently washed by adding the cells to 10 ml of cold (4°C) X-VIVO (Lonza) containing 10% AB serum per 10 × 10^6^ cells, in a drop-wise fashion. PBMCs were then washed again with 10 ml of cold (4°C) X-VIVO containing 1% AB serum per 10 × 10^6^ cells.

### T cell subset purification by cell sorting and DNA isolation.

CD4^+^ T cells (RosetteSep Human CD4^+^ T Cell Enrichment Cocktail, STEMCELL Technologies) were washed and immediately incubated with antibodies against surface molecules (Supplemental Table 3) for 40 minutes at 4°C, washed, and followed by sorting on a BD FACSAria Fusion flow cytometer cell sorter) into X-VIVO medium (Lonza) containing 5% human AB serum (Sigma-Aldrich). In order to isolate DNA, sorted cell subsets were checked for purity and DNA was isolated using a DNA extraction reagent (QIAGEN).

### sjTREC assay.

The sjTREC assay was performed as described previously (8). A quantitative PCR assay was purchased from Sigma-Genosys for the sjTREC that arises through an intermediate rearrangement in the TCRD/TCRA locus in developing TCRαβ^+^ T lymphocytes. An assay for the gene encoding albumin was used to normalize the data. The following primers were used: sjTREC.F, TCGTGAGAACGGTGAATGAAG; sjTREC.R, CCATGCTGACACCTCTGGTT; sjTREC.P, FAM-CACGGTGATGCATAGGCACCTGC-TAMRA; Alb.F, GCTGTCATCTCTTGTGGGCTGT; Alb.R, ACTCATGGGAGCTGCTGGTTC; Alb.P FAM-CCTGTCATGCCCACACAAATCTCTCC-TAMRA. For each sample, 24 ng of DNA was incubated in duplicate with both primers (700 nM), probe (150 nM), and 12.5 μl TaqMan mastermix (Applied Biosystems) and processed using the Applied Biosystems 7900HT Fast Real-Time PCR System. sjTRECs were normalized to the albumin gene, representing cellular DNA, using the following formula: 2 ^(Ct^ ^[albumin]^^−^^Ct^ ^[sjTREC])^.

### T cell activation.

FACS-purified T cell subsets or total CD4^+^ T cells (RosetteSep) for cord blood samples and samples from MS patients in Figure 4D were stimulated with either anti–CD3/CD28 beads (Life Technologies) at 3 cells per bead overnight (for RNA expression analyses) or cell stimulation reagent (PMA and ionomycin, eBioscience) in the presence of protein transport inhibitors (eBioscience) for 6 hours at 37°C in 96-well U-bottom plates (for intracellular cytokine determinations). IL-8^+^ and IL-2^+^ T cells were identified with a staining panel shown in Supplemental Table 3.

### Microarray gene expression analysis.

Total RNA was prepared from cell subsets isolated by sorting using TRIzol reagent (Life Technologies). Single-stranded cDNA was synthesized from 200 ng of total RNA using the Ambion WT Expression kit (Ambion) according to the manufacturer’s instructions. Labeled cDNA (GeneChip Terminal Labeling and Hybridization Kit, Affymetrix) was hybridized to a 96 Titan Affymetrix Human Gene 1.1 ST array.

Power calculations to determine the sample size required were performed using the method of Tibshirani (43), using a reference dataset from the Affymetrix GeneST array (deposited in ArrayExpress (http://www.ebi.ac.uk/arrayexpress/, accession number E-MTAB-4852) using the TibsPower package (http://github.com/chr1swallace/TibsPower) in R. We chose 20 pairs to have a false discovery rate (FDR) close to zero while detecting a 5-fold change in gene expression in 20 genes with a false negative rate of 5% or a 2-fold change in gene expression in 20 genes with a false negative rate of 40%, at a significance threshold of 10^–6^.

Microarray gene expression log2 intensities were normalized using vsn2 (44). Analysis of differential expression (log2 intensities) was conducted pairwise between each cell subtype using paired *t* tests with limma (45). *P* values were adjusted using the Benjamini-Hochberg algorithm. Illustrative principal component analysis was performed on the union of the most differentially expressed genes in each pairwise comparison. Data are deposited with ArrayExpress, accession number E-MTAB-4853.

### NanoString and RNA-seq: sample preparation and data analysis.

See Supplemental Figure 7 for a description of the NanoString and RNA-seq experimental design. CR2^+^ and CR2^−^ naive and memory cell subsets isolated by sorting CD4^+^ T cells (RosetteSep) from 4 donors were pelleted directly or following activation and lysed in QIAGEN RLT buffer and frozen at −80°C. To extract RNA, lysates were warmed to room temperature and vortexed. The RNA was extracted using a Zymo Research Quick-RNA MicroPrep kit following the manufacturer’s recommended protocol including on-column DNA digestion. RNA was eluted in 6 μl of RNase-free water. NanoString RNA expression analysis was performed using the Human Immunology v2 XT kit (NanoString Technologies), and 5 μl of RNA (5 ng/μl) was used per hybridization and set up following the recommended XT protocol. Hybridization times for all samples were between 16 and 20 hours. A NanoString Flex instrument was used and the Prep Station was run in high sensitivity mode and 555 fields of view were collected by the Digital Analyser. For RNA-seq analysis, 10 μl of RNA (8 ng/μl) was processed by AROS Applied Biotechnology using the Illumina TruSeq Access method that captures the coding transcriptome after library prep.

Raw NanoString expression measurements were normalized with application of NanoString software (nSolver 2.5). Subsequently, a paired differential expression analysis was carried out using DESeq2 v1.12.3 (46), with preset size factors equal to 1 for all samples. Analyses were performed using an FDR of 0.05%. A missing FDR is reported for genes that were found to contain an expression outlier by DESeq2 Cook’s distance-based flagging of *P* values. NanoString data are deposited with ArrayExpress, accession number E-MTAB-4834.

RNA-seq yielded on average 35.9 million paired-end reads per library. Maximum likelihood transcript read count estimates for each sample were obtained with Kallisto v0.42.5 (47), using Ensembl Release 82 (48) as a reference transcriptome. Gene expression estimates were derived by aggregating all their constituent transcript read counts, which were then employed to perform a paired differential expression analysis using limma v3.28.5 (45). For RNA-seq data, limma estimates the mean-variance relationship of log-counts and a precision weight for each gene observation (replicate) is generated to moderate standard errors (49). Analyses were performed using an FDR of 0.05%. A missing FDR is reported for genes that did not contain at least 2 counts per million (CPM) in at least 2 samples. Data from RNA-seq have been deposited with the European Genome-phenome Archive, http://www.ebi.ac.uk/ega/, accession number EGAS00001001870.

### Statistical analysis of flow cytometry data interrogating T cell subsets.

Statistical analyses of flow cytometry data were performed and presented using Prism 5 software (GraphPad) unless otherwise stated. Comparisons between cell subsets were performed using a paired *t* test unless otherwise stated. *P* less than 0.05 was considered significant; error bars show the SD of the samples at each test condition.

A nonparametric method, LOESS (50), performed with R software (http://www.R-project.org) was used to analyze some of the datasets The gray zones define a 95% confidence interval for each regression line.

## Author contributions

MLP, JAT, and LSW codesigned the study, evaluated the results, and cowrote the manuscript. ARG and CW contributed to the design of the study, evaluated the results, and edited the manuscript. CW performed statistical analysis of microarray data. ARG performed statistical analysis of NanoString and RNA-seq data. MLP, RCF, DBR, DJS, MM, JB, NS, XCD, SM, SD, AJ Cutler, and CSL performed experiments. HES coordinated sample collection and processing. NMW coordinated sample and data management. AJ Coles and JLJ codesigned and cosupervised the alemtuzumab study and edited the manuscript. DBD edited the manuscript. DBD, FWL, LSW, and JAT managed acquisition of samples from normal healthy volunteers as detailed in the Methods section.

## Supplementary Material

Supplemental data

Supplemental data 2

Supplemental data 3

Supplemental data 4

Supplemental data 5

Supplemental table 1

Supplemental table 2

## Version 1 08/17/2017

Electronic publication

## Version 2 12/14/2023

There were two broken hyperlinks that were fixed
