# Supplementary material for: Neonatal and adult recent thymic emigrants produce IL-8 and express complement receptors CR1 and CR2
Source: JCI Insight. 2017 Aug 17;2(16):e93739. doi: 10.1172/jci.insight.93739 (PMC5621870; doi:10.1172/jci.insight.93739)
Supplement: Supplemental data [file jciinsight-2-93739-s019.pdf]

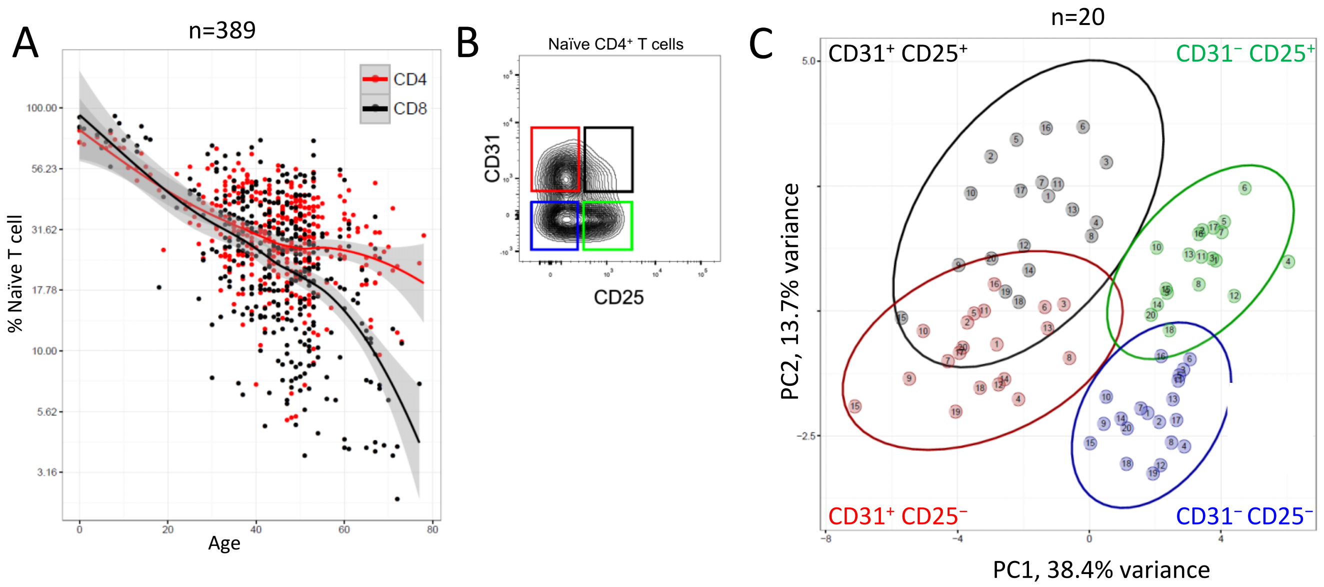

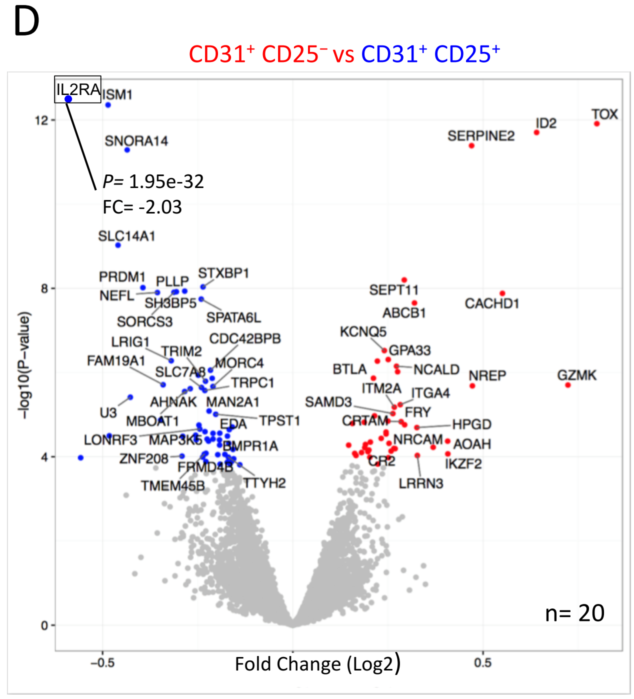

Supplemental Figure 1. Gene expression profiling of four naïve CD4⁺ T cell subsets identify age-related molecular signatures. (**A**) Frequency of naïve CD4⁺ and CD8⁺ naïve T cells out of total CD4⁺ and CD8⁺ T cells, respectively, as a function of age (n=389, 371, 15 and 3 from cohorts 1-3, respectively). (**B**) FACS sorting strategy of naïve CD4⁺ T cells stratified by expression of CD31 and CD25. (**C**) Principal component analysis (PCA) based on differentially expressed genes amongst four naïve CD4⁺ T cell subsets stratified by surface expression of CD31 and CD25 (subsets sorted from 20 donors, cohort 1). (**D**) Volcano plot representing differences in gene expression between CD31⁺ CD25⁻ and CD31⁺ CD25⁺ naïve CD4⁺ T cells; genes higher in CD31⁺ CD25⁻ (red) versus those higher in CD31⁺ CD25⁺ (blue) naïve CD4⁺ T cells; *P* value (*P*) and Fold Change (FC) for *IL2RA* (encoding CD25) are noted since the values fall outside of the graph boundaries (n=20, cohort 1).

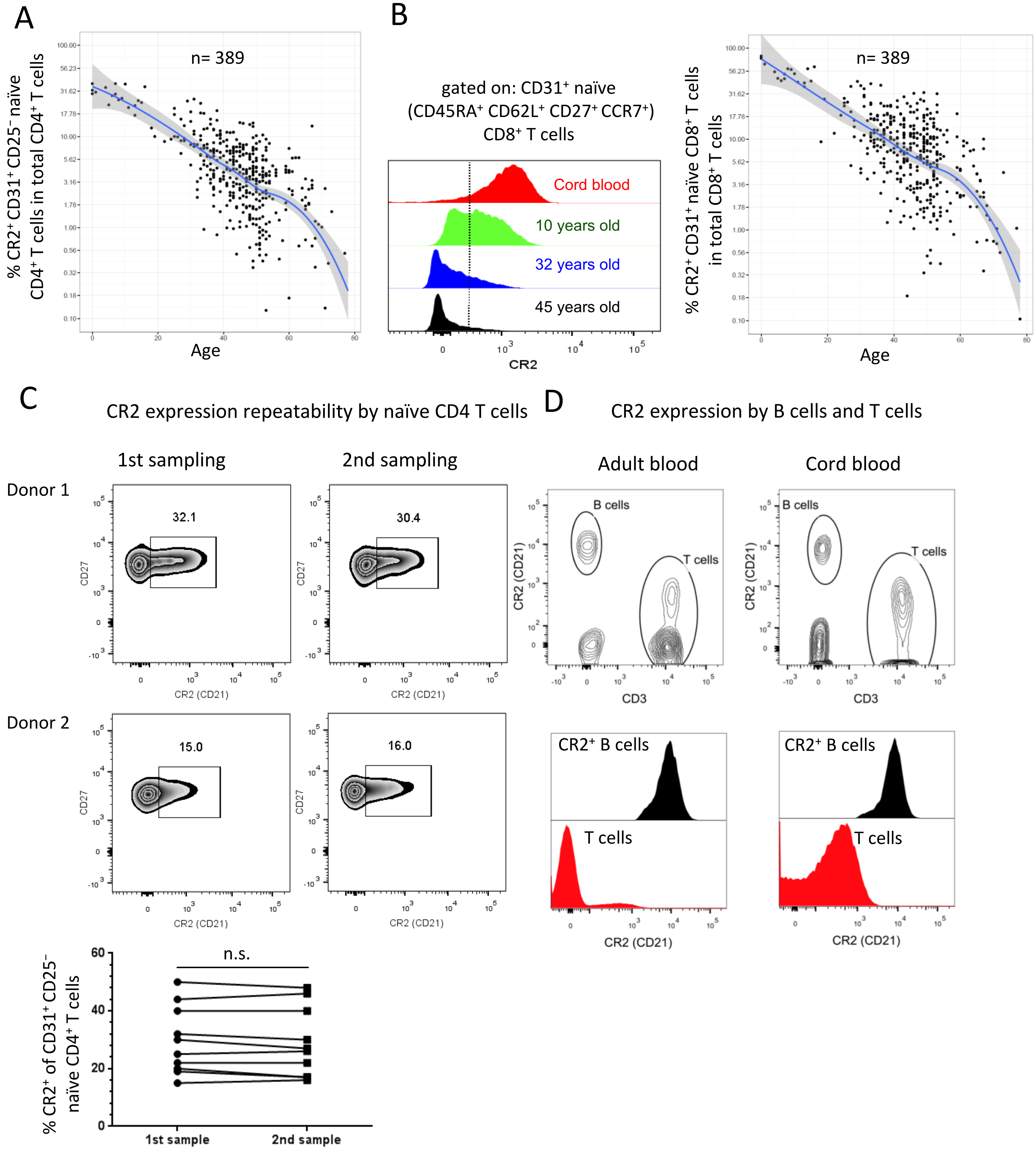

Supplemental Figure 2. CR2 protein expression in recent thymic emigrants. (**A**) The frequency of CD31⁺ CD25⁻ naïve CD4⁺ T cells out of total CD4⁺ T cells that are CR2⁺ as a function of age (n=389; 371, 15 and 3 donors from cohorts 1-3, respectively) . (**B**) Representative examples of CR2 expression on naïve CD8⁺ T cells and the frequency of CD31⁺ naïve CD8⁺ T cells out of total CD8⁺ T cells that are CR2⁺ as a function of age (see Supplemental Figure 5B for gating strategy of naïve CD8⁺ T cells). **(C)** Two representative examples and compiled data (paired t test) from ten donors (a subset of the 371 immunophenotyped donors in cohort 1) assessed for CR2 on CD31⁺ CD25⁻ naïve CD4⁺ T cells on two occasions (11 to 17 months between samples). **(D)** A comparison of cell-surface CR2 expression on T cells and CR2⁺ B cells (representative examples from cohorts 1 and 2).

A

Patient ID	CD4 T cell total count at Baseline million/ml	CD4 T cell count reconstituted at 12 month million/ml [% of Baseline]	Naïve CD4 T cell count at Baseline million/ml	Naïve CD4 T cell count reconstituted at 12 months million/ml [% of Baseline]	CR2+ CD4 naïve T cells at Baseline % of CD31+ CD25- cells
MS-6	1.2	0.18 (15%)	0.63	0.034 (5.4%)	18%
MS-2	0.68	0.14 (21%)	0.14	0.0034 (2.4%)	17%
MS-1	1.0	0.17 (17%)	0.60	0.11 (18%)	54%
MS-3	0.64	0.20 (31%)	0.32	0.11 (34%)	40%
MS-4	1.3	0.17 (13%)	0.55	0.071 (13%)	32%
MS-8	1.2	0.37 (31%)	0.45	0.14 (31%)	30%
MS-7	0.79	0.28 (35%)	0.28	0.18 (64%)	41%
MS-5	0.85	0.38 (45%)	0.12	0.072 (60%)	29%

B

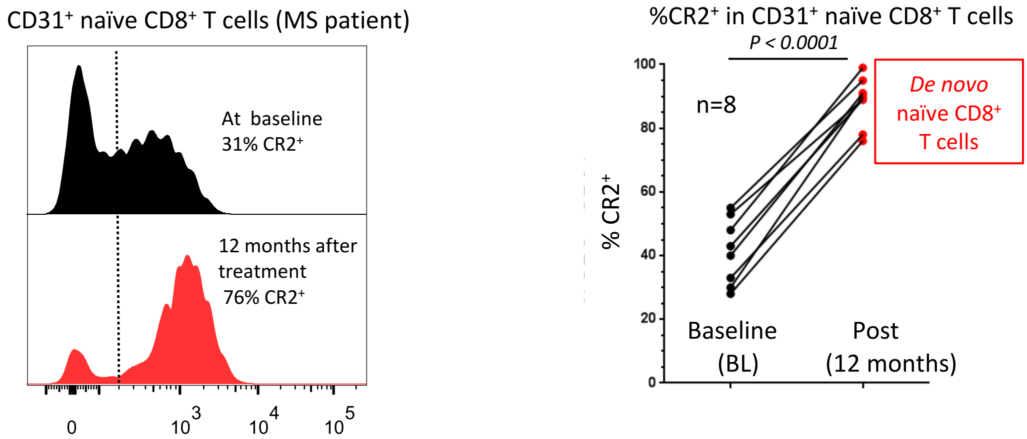

C

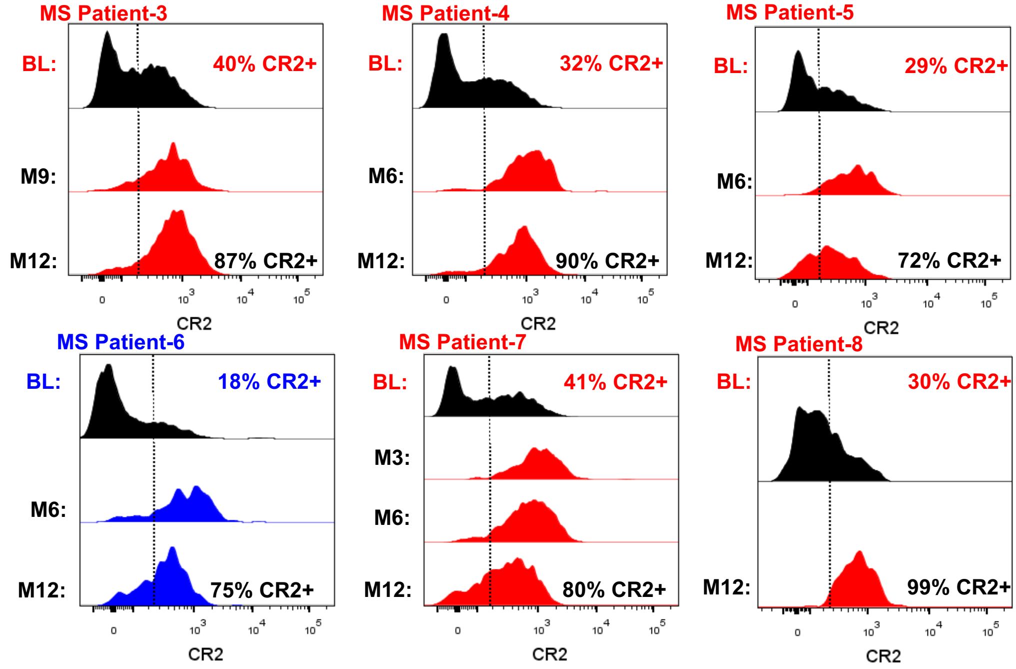

Supplemental Figure 3. Reconstitution of naïve T cells following lymphocyte depletion in MS patients. (**A**) Total and naïve CD4⁺ T cell (CD45RA⁺ CD62L⁺) counts at baseline (BL), the proportions reconstituted 12 months after depletion and the proportion of CR2⁺ cells in the CD31⁺ CD25⁻ naïve CD4⁺ T cell subset at BL are shown for eight patients. (**B**) Representative example and summary of CR2 expression on CD8⁺ naïve T cells at BL and 12 months after reconstitution. (**C**) CR2 expression profiles of CD31⁺ CD25⁻ naïve CD4⁺ T cells at BL and time points after lymphocyte depletion (see **Figure 3** for two other patients). CR2⁺ cell frequencies in the CD31⁺ CD25⁻ naïve CD4⁺ T cell subset are shown at BL and month 12. Profiles depicted in red and blue denote patients with good and poor, respectively, naïve T cell reconstitution at 12 months following depletion (see **Figure S3A** above).

A

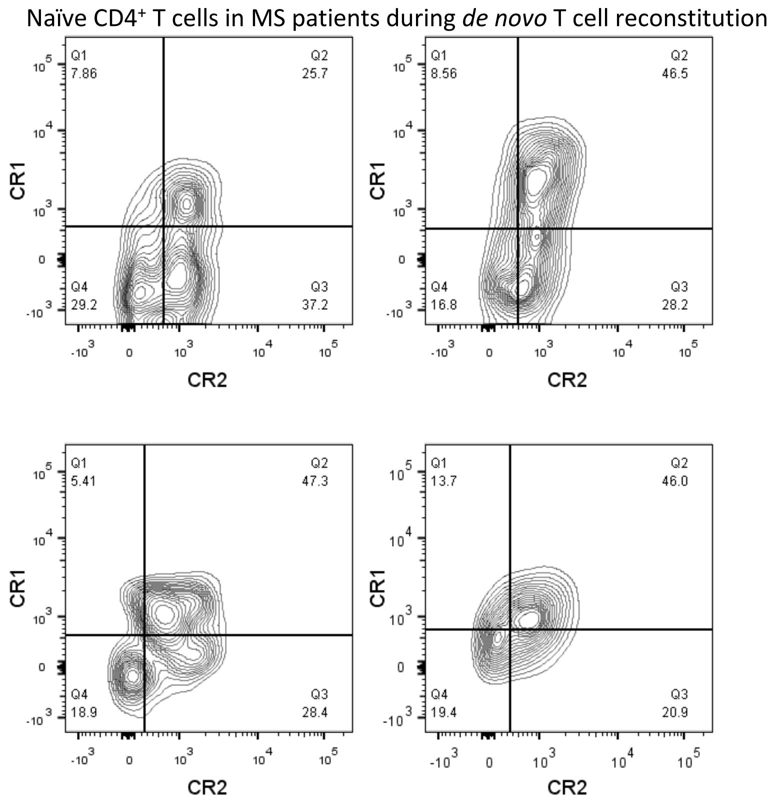

Supplemental Figure 4. CR2 and CR1 are co-expressed on naïve T cells. (**A**) CR1 and CR2 expression on CD31⁺ CD25⁻ naïve CD4⁺ T cells in MS patients (n=4) 6 to 12 months post-alemtuzumab. (**B**) Representative example and summary analysis of cell-surface CR1 expression on CR2⁺ and CR2⁻ CD31⁺ naïve CD8⁺ T cells (n=34, age range 0-67 yr, cohorts 1-3, paired t test). **(C)** CR2 and CR1 expression on CD31⁺ naïve CD8⁺ T cells in MS patients (n=3) 6 to 12 months post-alemtuzumab. **(D)** Number of CR2 and PTK7 transcripts in CR2⁺ and CR2⁻ naïve CD4⁺ T cells (CD31⁺CD25⁻) based on RNA-seq analysis, counts per million reads (cpm); (n=4, age range 30-44, cohorts 1 and 3, paired t test). **(E)** CR2 is lost from the surface of naïve T cells after 6h of activation with PMA and ionomycin (example from cord blood, cohort 3).

B

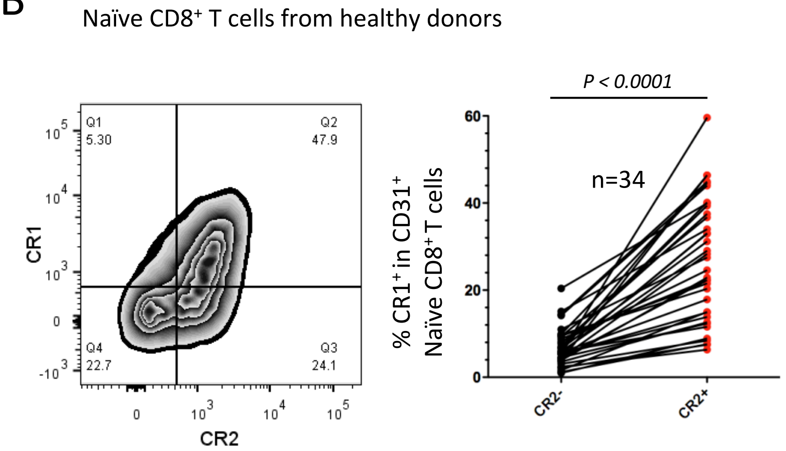

D

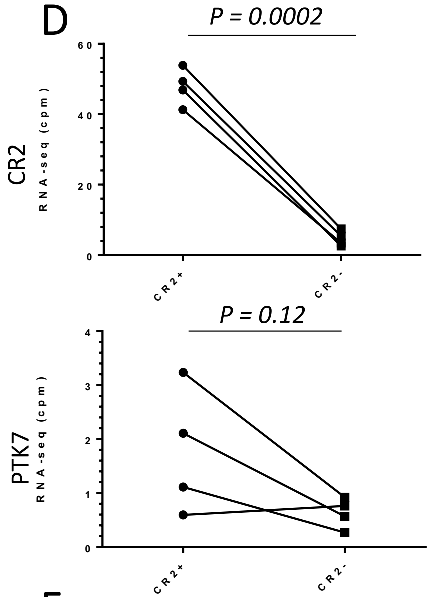

C

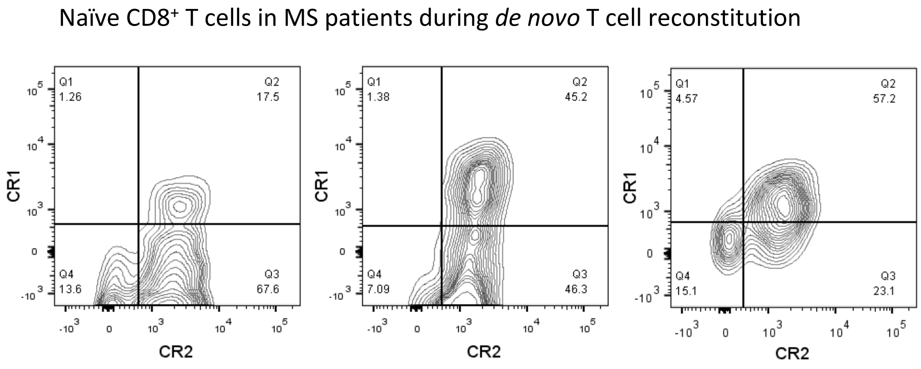

E

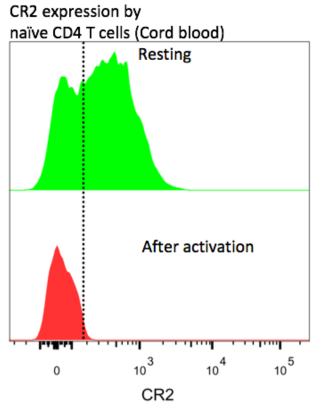

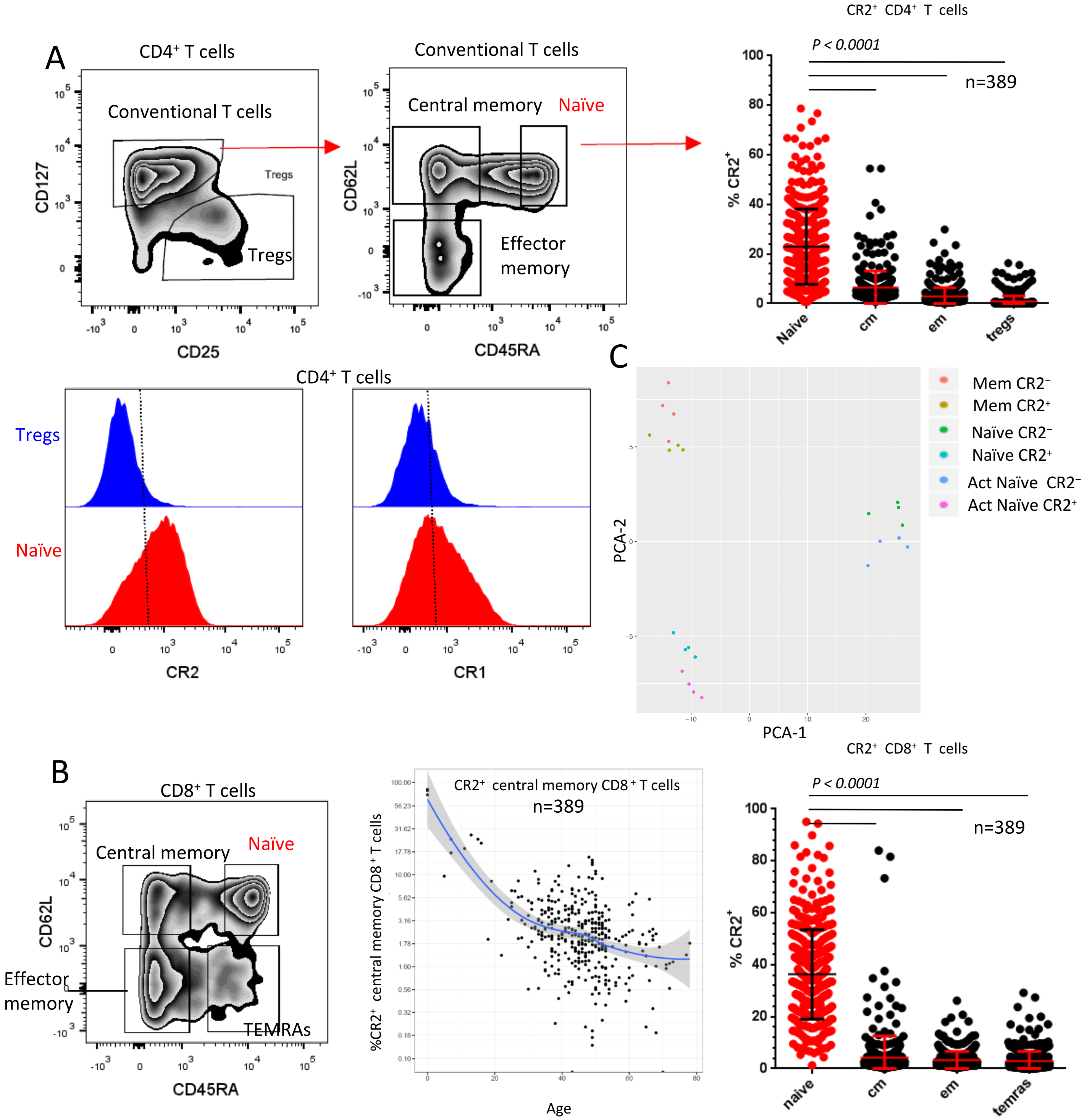

Supplemental Figure 5. CR2 is expressed by subsets of CD4+ and CD8+ memory T cells. (**A**) Representative gating of Tregs and naïve, central memory and effector memory CD4+ T cells and the distribution of CR2+ cells within each of these subsets (n=389; 371, 15 and 3 from cohorts 1-3, respectively, paired t tests for the comparisons indicated). Representative examples of CR1 and CR2 expression on naïve CD4+ T cells and CD4+ Tregs from a donor 10 years of age. (**B**) Representative gating of naïve and memory CD8+ T cell subsets. CR2 expression on central memory CD8+ T cells as a function of age. Distribution of CR2+ cells within naïve and memory CD8+ T cell subsets (mean ± SD, paired t tests for comparisons) (n=389; 371, 15 and 3 from cohorts 1-3, respectively). (**C**) Principal component analysis (PCA) based on gene expression (NanoString) of CR2+ and CR2− naïve and central memory CD4+ T cells *ex vivo* and activated CR2+ and CR2− naïve CD4+ T cells (n=4 adult donors from cohorts 1 and 3).

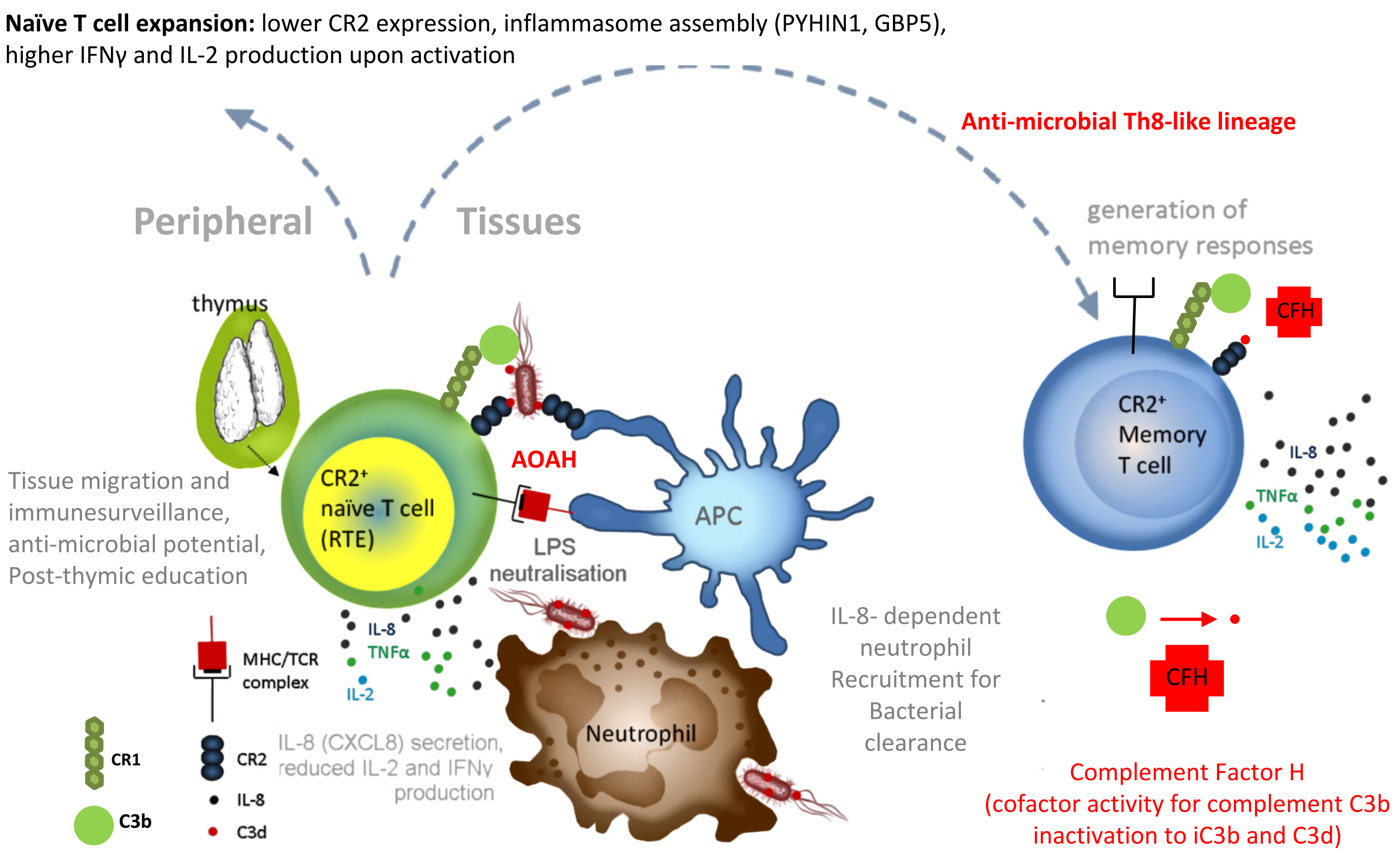

Differentially expressed genes
(Higher expression in CR2$^+$ vs CR2$^-$ naïve T cells *ex vivo*)

AOAH, ADA, CACHD1, DACH1, FCGRT, ITGA4, ITGA6, TCF4, TLR1, LRRN3, TOX, IKZF2 (HELIOS)

Differentially expressed genes
(Higher expression in CR2$^+$ vs CR2$^-$ memory T cells *ex vivo*)

ANK1, TNFSF13B (BAFF), CFH, CD79A, ZBTB16, IL2RA (CD25), NT5E (CD73), IL7R

Differentially expressed genes shared between CR2$^+$ naïve and CR2$^+$ central memory CD4$^+$ T cells vs CR2$^-$ naïve and CR2$^-$ central memory CD4$^+$ T cells *ex vivo*

CR1, CR2	Complement receptors, regulate lymphocyte activation, recognition of microbial products; C3b inactivation
ADAM23	Metalloproteinase, binds to αVβ3 integrin, regulates cell-cell and cell-matrix interactions
ARHGAP32 (RICS)	Rho GTPase-activating protein 32 neuron-associated GTPase-activating protein, regulates cell morphology
DST	Dystonin, Bullous pemphigoid antigen; plakin protein family of adhesion junction plaque proteins, potentially aids in migration through tissues
PLXNA4	Plexin A4 binds to neuropilin 1 (Nrp1) and neuropilin 2 (Nrp2), regulation of cell morphology and migration
TNFAIP3	zinc finger protein and ubiqitin-editing enzyme, attenuates TNF signalling, inhibits NF-kappa B activation as well as inhibits TNF-mediated apoptosis
BCL2	Anti-apoptotic protein
CISH	CISH controls T cell receptor (TCR) signalling and suppressor of cytokine signalling (SOCS), involved in anti-bacterial responses.
SOCS3	Suppressor of cytokine signalling 3, inhibition of JAK/STAT activation, regulator of infection and inflammation.
ZNF462	Transcription factor

CTLA4, GBP5, GZMK, PYHIN1, SLAM6 ↓

Supplemental Figure 6. Hypothetical model of molecular and cellular interactions occurring in CR2^{+} T cells based on gene and protein expression.

Gene names shown in the first two boxes are some of the differentially expressed genes having higher expression in either CR2^{+} naïve or CR2^{+} memory CD4^{+} T cells versus their CR2^{-} counterparts. Genes that share their expression patterns in both CR2+ naïve and memory cells are listed in the larger boxes (genes with higher expression in red, genes with lower expression in blue). We have discovered that CR2^{+} T cells express a selection of genes that should give them the potential to migrate to tissue including the gut-homing integrin alpha4 (*ITGA4*) (1) and integrin alpha6 (*ITGA6, CD49f*), a hemidesmosomal component important for tissue homing (2, 3). We hypothesize that in the presence of appropriate danger signals and tissue-based antigen-presenting cells, CR2^{+} T cells respond to pathogens by mediating IL-8-dependent neutrophil recruitment and differentiating into memory cells, some of which maintain the ability to secrete IL-8. This scenario of priming naïve T cells within tissues is supported by the presence in children of a higher proportion (>35%) of effector memory CD4^{+} and CD8^{+} T cells in jejunum, ileum and lung tissue samples as compared to lymph nodes (<10% Teff) (4). CR2^{+} naïve and memory T cells share the expression of other genes that are involved in cell adhesion and may enhance the migratory properties of the T cells through the basal lamina: *DST* that encodes an adhesion junction protein anchoring intermediate filaments to hemidesmosomes (5, 6), *ARHGAP32* (*RICS*) that regulates neuronal cell morphology and movement (7) and *PLXNA4* that is also involved in nerve fiber guidance (8).

1. Sun H, Liu J, Zheng Y, Pan Y, Zhang K, Chen J. Distinct chemokine signaling regulates integrin ligand specificity to dictate tissue-specific lymphocyte homing. *Dev Cell.* 2014;30(1):61-70.

2. Qian H, Tryggvason K, Jacobsen SE, Ekblom M. Contribution of alpha6 integrins to hematopoietic stem and progenitor cell homing to bone marrow and collaboration with alpha4 integrins. *Blood.* 2006;107(9):3503-3510.

3. Sonawane M, Martin-Maischein H, Schwarz H, Nusslein-Volhard C. Lgl2 and E-cadherin act antagonistically to regulate hemidesmosome formation during epidermal development in zebrafish. *Development.* 2009;136(8):1231-1240.

4. Thome JJ, et al. Early-life compartmentalization of human T cell differentiation and regulatory function in mucosal and lymphoid tissues. *Nat Med.* 2016;22(1):72-77.

5. Koster J, Geerts D, Favre B, Borradori L, Sonnenberg A. Analysis of the interactions between BP180, BP230, plectin and the integrin alpha6beta4 important for hemidesmosome assembly. *J Cell Sci.* 2003;116(Pt 2):387-399.

6. Kunzli K, Favre B, Chofflon M, Borradori L. One gene but different proteins and diseases: the complexity of dystonin and bullous pemphigoid antigen 1. *Exp Dermatol.* 2016;25(1):10-16.

7. Okabe T, et al. RICS, a novel GTPase-activating protein for Cdc42 and Rac1, is involved in the beta-catenin-N-cadherin and N-methyl-D-aspartate receptor signaling. *J Biol Chem.* 2003;278(11):9920-9927.

8. Kong Y, et al. Structural Basis for Plexin Activation and Regulation. *Neuron.* 2016;91(3):548-560.

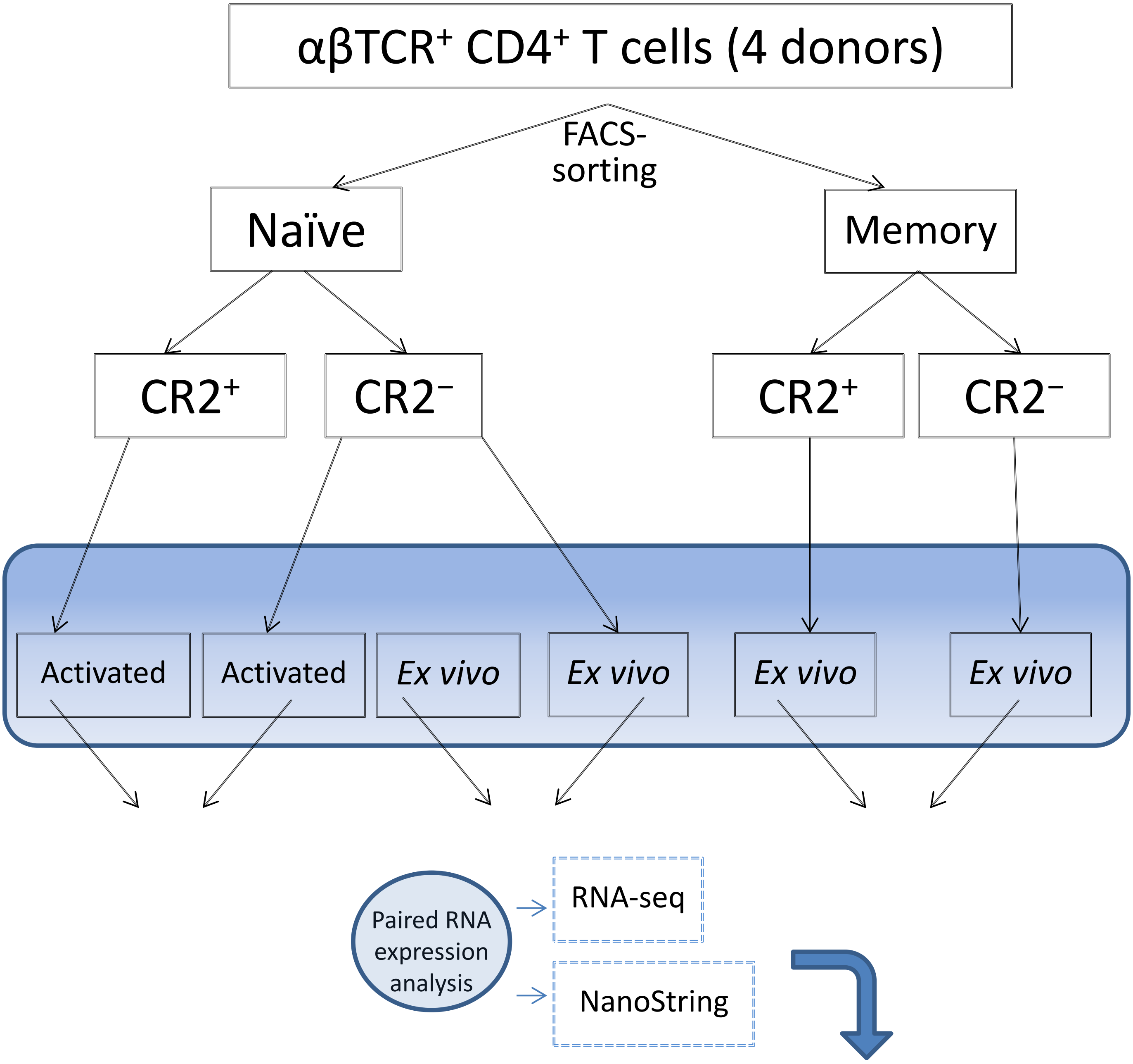

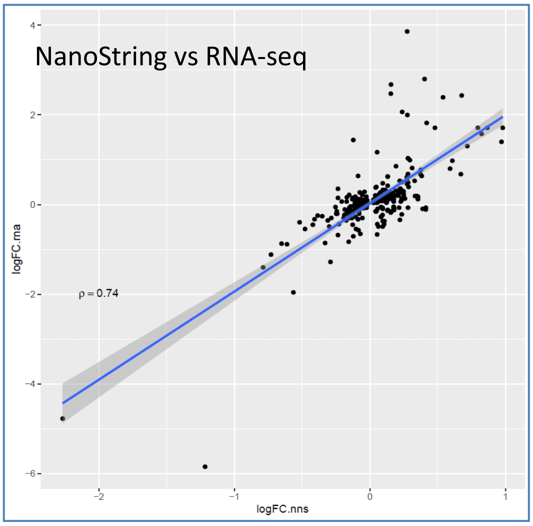

Supplemental Figure 7. Design of the RNA analysis experiment performed on naïve CR2⁺ and CR2⁻ and central memory CR2⁺ and CR2⁻ CD4⁺ T cells. Two RNA analysis methods, RNA-seq and NanoString (Human Immunology gene panel), were applied on paired RNA samples purified from FACS-sorted T-cell subsets from four donors. Fold change (Log2) differences obtained from RNA-seq and NanoString were highly correlated (example shown at the bottom right represents fold change between CR2⁺ and CR2⁻ central memory CD4⁺ T cells on both platforms).

Supplemental Table 1. Selected results of RNA analysis experiment (RNA-seq) performed on CR2⁺ and CR2⁻ naïve and memory CD4⁺ T cells *ex vivo.*

Gene ID		Naïve *ex vivo* CR2⁺	Naïve *ex vivo* CR2⁻		Memory *ex vivo* CR2⁺	Memory *ex vivo* CR2⁻	
**CR1**							
	Donor1	271.382*	27.106	FC**	1008.486	39.988	FC
	Donor2	162.204	12.753	10.204	825.464	22.576	27.027
	Donor3	180.323	9.47	Adj P value***	1374.156	37.313	Adj P value
	Donor4	367.677	56.653	0.001	619.707	37.049	0.00001
**CR2**							
	Donor1	53.892	7.447	FC	53.824	0.881	FC
	Donor2	41.293	3.413	10.204	33.583	0.231	58.824
	Donor3	46.893	2.554	Adj P value	55.151	1.041	Adj P value
	Donor4	49.316	5.371	0.001	21.741	0.52	0.001
**ZNF462**							
	Donor1	6.921	0.44	FC	31.936	4.507	FC
	Donor2	3.14	0.237	12.987	17.958	0.971	7.194
	Donor3	13.643	0.887	Adj P value	24.472	4.212	Adj P value
	Donor4	10.583	0.967	0.005	23.426	2.914	0.005
**CACHD1**							
	Donor1	46.55	31.59	FC	1.997	0.102	FC
	Donor2	40.215	20.102	1.757	1.395	0.37	n/a
	Donor3	33.138	13.265	Adj P value	0.423	0.098	Adj P value
	Donor4	45.642	29.65	0.037	2.467	1.041	n/a
**ADAM23**							
	Donor1	11.703	2.482	FC	94.556	20.536	FC
	Donor2	2.344	0.711	6.803	45.053	9.016	5.051
	Donor3	21.67	1.561	Adj P value	90.442	14.808	Adj P value
	Donor4	14.583	2.578	0.02	36.686	6.765	0.00001
**PLXNA4**							
	Donor1	12.262	2.242	FC	83.232	20.638	FC
	Donor2	3.562	0	6.024	20.666	1.618	4.926
	Donor3	21.494	3.866	Adj P value	59.546	7.258	Adj P value
	Donor4	25.493	3.867	0.02	36.034	11.188	0.014
**AOAH**							
	Donor1	19.157	9.009	FC	7.017	7.388	FC
	Donor2	64.166	22.709	2.155	14.26	9.385	1.185
	Donor3	80.594	25.749	Adj P value	12.827	13.116	Adj P value
	Donor4	129.859	88.895	0.046	46.509	32.628	0.512
**CFH**							
	Donor1	0.037	0.08	FC	47.235	18.842	FC
	Donor2	0.094	0.759	n/a	41.539	23.903	2.653
	Donor3	0	0	Adj P value	45.677	10.805	Adj P value
	Donor4	0	0	n/a	58.334	19.254	0.016
**DST**							
	Donor1	11.404	5.966	FC	49.574	20.875	FC
	Donor2	15.467	7.823	1.770	30.896	8.692	2.653
	Donor3	1.653	0.816	Adj P value	33.11	10.089	Adj P value
	Donor4	32.451	20.196	0.038	61.639	25.655	0.006
**ARHGAP32**							
(RICS)	Donor1	37.381	24.183	FC	50.943	19.79	FC
	Donor2	52.589	19.39	2.105	59.209	21.083	2.427
	Donor3	49.637	18.443	Adj P value	31.417	11.391	Adj P value
	Donor4	55.383	28.629	0.02	35.429	19.462	0.007
**PYHIN1**							
	Donor1	5.516	12.772	FC	30.121	47.477	FC
	Donor2	8.718	17.589	0.444	45.466	68.102	0.703
	Donor3	3.694	9.151	Adj P value	43.3	51.65	Adj P value
	Donor4	3.061	6.07	0.02	33.66	44.076	0.039

*Gene counts per million reads **FC= Fold change of CR2⁺ vs CR2⁻ ***Adj P value= Adjusted P value
See Methods for analysis of RNA-seq data.

Supplemental Table 2. Selected results of RNA analysis experiment (NanoString) performed on CR2$^+$ and CR2$^-$ naïve CD4$^+$ T cells after activation.

Gene ID		Naïve activated		
		CR2$^+$	CR2$^-$	
IL8				
	Donor1	2529*	889	FC**
	Donor2	69	24	2.797
	Donor3	140	52	Adj P value***
	Donor4	187	50	7.36E-21
IL2				
	Donor1	3937	20724	FC
	Donor2	9108	31986	0.307
	Donor3	16729	40604	Adj P value
	Donor4	11808	40884	3.52E-23
IL21				
	Donor1	3	56	FC
	Donor2	25	281	0.107
	Donor3	34	385	Adj P value
	Donor4	26	341	3.19E-52
IFNG				
	Donor1	9	63	FC
	Donor2	8	17	0.426
	Donor3	14	36	Adj P value
	Donor4	36	84	3.11E-04
LIF				
	Donor1	418	769	FC
	Donor2	319	778	0.489
	Donor3	305	622	Adj P value
	Donor4	301	632	3.70E-16
TNF				
	Donor1	945	1130	FC
	Donor2	641	762	0.898
	Donor3	1487	1353	Adj P value
	Donor4	1580	1914	4.06E-01
IL23A				
	Donor1	578	536	FC
	Donor2	248	248	1.123
	Donor3	463	317	Adj P value
	Donor4	441	430	4.68E-01
LTA				
	Donor1	1115	1013	FC
	Donor2	1182	1187	1.137
	Donor3	1857	1362	Adj P value
	Donor4	2217	1965	2.66E-01

*Normalized Counts
**FC=Fold change of CR2$^+$ vs CR2$^-$
***Adj P value=Adjusted P value

Supplemental Table 3. Antibody panels used for immunophenotyping and FACS-sorting.

Panel	Antigen	Fluorochrome	Clone ID	Manufacturer
CR2 Immunophenotyping	CD3	BV510	OKT3	BioLegend
	CD4	BUV398	SK3	BD Biosciences
	CD8	APC-Cy7	RPA-T8	BioLegend
	CD56	BV711	NCAM	BD Biosciences
	CD127	PE-Cy7	eBioRDR5	eBioscience
	CD25*	APC	M-A251+2A3	BD Biosciences
	CD45RA	BV785	HI100	BioLegend
	CD62L	BV605	DREG56	BD Biosciences
	CCR7	BV421	GO43H7	BioLegend
	CD27	AlexaFluor700	M-T271	BioLegend
	CD95	PerCP eFluor710	DX2	BioLegend
	CD31	FITC	WM59	eBioscience
	CR2 (CD21)*	PE	BU32+Ly4	BioLeg+BD Bio
CR1/CR2 Immunophenotyping	CD3	BV510	OKT3	BioLegend
	CD4	BUV398	SK3	BD Biosciences
	CD8	APC-Cy7	RPA-T8	BioLegend
	CD56	BV711	NCAM	BD Biosciences
	CD127	PE-Cy7	eBioRDR5	eBioscience
	CD25*	APC	M-A251+2A3	BD Biosciences
	CD45RA	BV785	HI100	BioLegend
	CCR7	BV605	GO43H7	BioLegend
	CD27	AlexaFluor700	M-T271	BioLegend
	CD95	PerCP eFluor710	DX2	eBioscience
	CD31	FITC	WM59	eBioscience
	CR2 (CD21)*	PE	BU32+Ly4	BioLeg+BD Bio
	CR1 (CD35)	BV421	E11	BD Biosciences
FACS sorting of four naïve CD4+ subsets defined by CD25 and CD31 expression using CD4+ TCRab+ cells enriched by negative selection (RosetteSep)	TCRab+	FITC	IP26	BioLegend
	CD4	AF700	RPA-T4	BioLegend
	CD25*	APC	M-A251+2A3	BD Biosciences
	CD127	PE-Cy7	eBioRDR5	eBioscience
	CD45RA	BV785	HI100	BioLegend
	CD31	PE	WM59	BioLegend
	CD62L	BV605	RPA-T8	BioLegend
	CCR7	Pacific Blue	GO43H7	BioLegend
	Viability Dye	eFluor780		eBioscience
FACS sorting of naïve and memory subsets defined by CR2 expression using CD4+ TCRab+ cells enriched by negative selection (RosetteSep)	CD4	AF700	RPA-T4	BioLegend
	CD25*	APC	M-A251+2A3	BD Biosciences
	CD127	PE-Cy7	eBioRDR5	eBioscience
	CD45RA	BV785	HI100	BioLegend
	CD62L	BV605	RPA-T8	BioLegend
	CD31	BV421	WM59	BioLegend
	CR2 (CD21)	PE	BU32	BioLegend
	Viability Dye	eFluor780		eBioscience
Cytokine production (IL-8 and IL-2) following activation	CD4	AF700	RPA-T4	BioLegend
	CD25*	APC	M-A251+2A3	BD Biosciences
	CD127	PE-Cy7	eBioRDR5	eBioscience
	CD45RA	BV785	HI100	BioLegend
	IL-2	BV510	MQ1	BioLegend
	IL-8	FITC	E8N1	BioLegend
	Viability Dye	eFluor780		eBioscience
PTK7 Immunophenotyping	PTK7/ CCK-4	APC/PE	188B	Miltenyi Biotec
	CD4	BUV398	SK3	BD Biosciences
	CD127	PE-Cy7	eBioRDR5	eBioscience
	CD25*	V421/APC	M-A251+2A3	BD Biosciences
	CD45RA	BV785	HI100	BioLegend
	CD62L	BV605	DREG56	BD Biosciences
	CD95	PerCP eFluor710	DX2	BioLegend
	CD31	FITC	WM59	eBioscience
	CR2 (CD21)*	PE	BU32+Ly4	BioLeg+BD Bio

*Two clones each of anti-CD25 and anti-CR2 were used to enhance detection.
